# *Comamonadaceae* OTU as a Remnant of an Ancient Microbial Community in Sulfidic Waters

**DOI:** 10.1007/s00248-018-1270-5

**Published:** 2018-10-19

**Authors:** Edyta Deja-Sikora, Marcin Gołębiewski, Agnieszka Kalwasińska, Arkadiusz Krawiec, Przemysław Kosobucki, Maciej Walczak

**Affiliations:** 1 Interdisciplinary Center for Modern Technologies, Nicolaus Copernicus University, Wilenska 4, 87-100 Toruń, Poland; 2 Department of Environmental Microbiology and Biotechnology, Faculty of Biology and Environmental Protection, Nicolaus Copernicus University, Lwowska 1, 87-100 Toruń, Poland; 3 Chair of Plant Physiology and Biotechnology, Faculty of Biology and Environmental Protection, Nicolaus Copernicus University, Lwowska 1, 87-100 Toruń, Poland; 4 Department of Geology and Hydrogeology, Faculty of Earth Sciences, Nicolaus Copernicus University, Lwowska 1, 87-100 Toruń, Poland; 5 Department of Food Analysis and Environmental Protection, Faculty of Chemical Technology and Engineering, UTP University of Science and Technology, Seminaryjna 3, 85-326 Bydgoszcz, Poland

**Keywords:** Sulfidic waters, Intraterrestrial microbiome, Microbial diversity, Sulfate-reducing bacteria (SRB), Sulfur-oxidizing bacteria (SOB), Sulfur cycle

## Abstract

Intraterrestrial waters harbor microbial communities being extensively studied to understand microbial processes underlying subsurface ecosystem functioning. This paper provides the results of an investigation on the microbiomes of unique, subsurface sulfidic waters associated with Upper Jurassic, Cretaceous, and Miocene sediments. We used high-throughput 16S rDNA amplicon sequencing to reveal the structure of bacterial and archaeal communities in water samples differing in sulfide content (20–960 mg/dm^3^), salinity (1.3–3.2%), and depth of extraction (60–660 m below ground level). Composition of the bacterial communities strongly varied across the samples; however, the bacteria participating in the sulfur cycle were common in all sulfidic waters. The shallowest borehole water (60 m bgl) was dominated by sulfur-oxidizing *Epsilonproteobacteria* (*Sulfurimonas*, *Sulfurovum*). In the waters collected from greater depths (148–300 m bgl), the prevalence of *Betaproteobacteria* (*Comamonadaceae*) and sulfate/sulfur-reducing *Deltaproteobacteria* (*Desulfopila*, *Desulfomicrobium*, *MSBL7*) was observed. Sulfate reducers (members of *Clostridia*: *Candidatus Desulforudis*) were the most abundant bacteria in the deepest borehole water (660 m bgl). Out of 850 bacterial OTUs, only one, affiliated with the *Comamonadaceae* family, was found abundant (> 1% of total bacterial sequences) in all samples. Contribution of *Archaea* to the whole microbial communities was lower than 0.5%. Archaeal communities did not differ across the samples and they consisted of *Halobacteriaceae*. Out of 372 archaeal OTUs, five, belonging to the four genera *Natronomonas*, *Halorubrum*, *Halobellus*, and *Halorhabdus*, were the most numerous.

**Electronic supplementary material:**

The online version of this article (10.1007/s00248-018-1270-5) contains supplementary material, which is available to authorized users.

## Introduction

Microbial communities of terrestrial, subsurface hydrosphere are subject to extensive studies [1–4]. They greatly contribute to the Earth’s total biomass [5]. Special interest focused on ecosystems thriving underground results from our fragmentary knowledge on microorganisms capable of inhabiting deep ecological niches like fracture fluids. Many investigations of intraterrestrial biosphere revealed that cells remain active in spite of extreme environmental conditions, for instance high temperature and pressure or low content of nutritional compounds and energy sources [6–8]. These microorganisms are involved in biogeochemical processes driving carbon, nitrogen, and sulfur cycling [8–11]. Thus, exploring microbial diversity of intraterrestrial aquifers may improve our understanding of microorganism-mediated redox reactions influencing water chemistry, as well as may help to identify keystone phylogenetic groups related to particular water qualities.

Sulfide-rich mineral waters are recognized in SE Poland, in the vicinity of Busko-Zdrój town, located within the northern part of the Carpathian Foredeep. The hydrogeological structure of Busko-Zdrój area is complicated. The Miechow Trough, which is found therein, consists of Quaternary, Neogenic, Cretaceous, Jurassic, and Triassic multiaquifer [12, 13]. Sulfidic waters of Cl-Na, H_2_S, I, and F (*Br*, *HBO*_*2*_) type and mineralization in the range of 12.5–32 g/dm^3^ are extracted from the Cretaceous, Neogenic, and Jurrasic strata. It is hypothesized that primary sulfates in waters originated from dissolution of Badenian gypsum, while salinity was the effect of washing out the salt deposits during the infiltration period. Currently observed high concentration of sulfides is expected to result from microbial redox activities in this anoxic environment [14–16]. The isotopic composition (^*18*^*O*, ^*2*^*H*, ^*3*^*H*, ^*4*^*He*, ^*40*^*Ar*) suggests that sulfidic waters originated from the meteoric water infiltration, which occurred in the last interglacial period and earlier, in the warm Pre-Pleistocene climates [17, 18].

Sulfide-rich mineral waters from Busko-Zdrój are classified as fossils and their exploitation is under the control of the Polish Geological Institute (PGI). Due to their unique properties, sulfidic waters are well known and valued curative agents commonly used in balneotherapy for baths, inhalations, and drinking. They contain at least 1 mg of sulfur per dm^3^, found as hydrogen sulfide (H_2_S), hydrosulfide ions (HS^−^), polysulfides (H_2_S_*x*_, *x* = 2–6), and thiosulfate ions (S_2_O_3_^2−^) [19]. Additionally they are enriched in iodide (> 1 mg/dm^3^) and fluoride (> 1 mg/dm^3^) in doses exceeding pharmacodynamic thresholds [19, 20]. These groundwaters, due to their wide therapeutic applications, are under regular and strict sanitary control in order to preclude pathogenic microbial species contamination; however, their microbiome still remains unknown.

Microbial community compositions of sulfide-rich terrestrial and subterrestrial waters were investigated with both classical (culture-dependent) and molecular (culture-independent) methods [3, 8, 14, 15, 21–23]. A few microbial diversity analyses of several Carpathian sulfidic springs done so far were based on microscopic observations and gave only the superficial view of microbial life [14, 24]. They showed that sulfide-rich waters are abundantly inhabited by diverse sulfur-oxidizing bacteria (SOBs), that form consortia consisting either of the genera *Chlorobium*, *Chromatium* and *Thiothrix*, or *Beggiatoa* and *Thiothrix* [14]. More detailed data come from the molecular studies of bacterial and archaeal microbiome of other sulfur-rich environmental niches like euxinic lakes and marine sulfidic redoxcline waters [11, 23, 25–28]. However, environmental conditions in sulfidic springs, marine waters, or euxinic lakes differ dramatically from those found underground in terms of access to light, oxygen, and organic matter fluxes. Therefore, the structure of communities found in the aforementioned habitats cannot be used for simple inference on microbial communities in subsurface sulfide-rich water.

Only scarce information on microbiome of intraterrestrial sulfidic waters is available in the literature. Pimenov and coworkers investigated microorganisms indigenous to sulfide-rich water (ca. 240 mg H_2_S/dm^3^) associated with early Permian deposits of limestone and dolomites [22]. Sulfate-reducing bacteria (SBR), obtained as enrichment cultures, were identified by small-scale 16S rRNA gene cloning and sequencing. The results showed predomination of sulfate-reducing bacteria (SRB) group 6 (*Desulfovibrio-Desulfomicrobium*) [22]; however, no other methods were used to assess microbiome inhabiting original water.

The objective of the present work was to reveal the bacterial and archaeal community structure in subsurface sulfidic waters using high-throughput 16S rDNA amplicon sequencing method. We wanted to establish the core bacterial and archaeal OTUs (common for all samples) of the waters under study and check if potential differences in community composition are related to site-specific conditions like water chemistry, H_2_S content, and depth of extraction. Finally, our aim was to recognize the microbial groups potentially involved in sulfur cycling, since they might strongly influence the properties of sulfide-rich waters.

## Materials and Methods

### Water Sample Collection

Samples of sulfidic waters were collected in August 2014, from five different boreholes - 4B, 17, C1, Dobrowoda, and Welnin, located within and nearby Busko-Zdrój town (Supplementary Fig. S1). Detailed geographical coordinates of boreholes are as follows: Busko 4B (50° 27′ 19.10″ N, 20° 43′ 06.00″ E), Busko 17 (50° 27′ 16.50″ N, 20° 43′ 23.20″ E), Busko C1 (50° 28′ 10.23″ N, 20° 44′ 18.37″ E), Dobrowoda (50° 24′ 07.15″ N, 20° 47′ 36.45″ E), and Welnin (50° 20′ 23.10″ N, 20° 55′ 06.30″ E). The distances between collection sites are given in Supplementary Table S1. Five-liter water samples were taken into sterilized glass bottles (Schott AG, Mainz, Germany). Prior to the sampling, stagnant water was pumped out and valve outlet was flame-sterilized. Bottles were completely filled with water, avoiding air bubbles, then tightly sealed and transported directly to the laboratory. Water samples were stored at 8 °C until analyzed that started within 24 h since the sampling time.

### Hydrogeological Characteristics

Busko 4B, Busko 17, and Busko C1 sampling sites are located close to each other. Dobrowoda and Welnin are more distant, with the outermost Welnin lying approximately 19 km from Busko C1 (Table 1). Boreholes differ in depth and geological structure of water-bearing strata. The shallowest Busko 4B reaches the depth of 60 m below ground level (bgl), while Busko C1 is over tenfold deeper, reaching 660 m bgl (Table 1).

**Table 1:** Physicochemical and geological parameters of waters

Parameter	Busko 4B	Busko 17	Busko C1	Dobrowoda	Wełnin
Depth^a^ [m bgl]	60	148	663	300	170
pH	7.03	7.03	7.8	6.9	6.6
Eh [mV]	− 349	− 360	− 375	− 358	− 209
EC^b^ [mS/cm]	21.7	22	17.3	22.4	48.7
Temperature [°C]	12.4	12.7	22.8	16.3	13.5
Cations [mg/dm^3^]	4691.1	4643.5	4654.3	4830.5	11,478.3
Na^+^	3949.6	3875	4166.7	3897.3	9303.3
K^+^	100.8	99.9	80.7	105.1	189.1
Ca^2+^	376.4	395.4	232.5	483.9	1035
Mg^2+^	226	230.9	155.6	291.6	873.2
NH_4_^+^	16.7	23.5	18.8	35.9	39.6
Fe^2+^	< 0.1	< 0.1	< 0.1	< 0.1	0.3
Anions [mg/dm^3^]	8531.2	8377.6	8056.5	9069.4	20,975.4
Cl^−^	6156	5938	5743	6205	17,643
SO_4_^2−^	1918	1962	1893	2394	2303
HCO_3_^−^	431.4	452.3	408.6	456.2	944.3
NO_3_^−^	0.57	0.62	0.55	0.63	63.23
PO_4_^3−^	BDL^c^	BDL	BDL	BDL	BDL
Br^−^	22.2	21.7	9.3	10.2	68.2
I^−^	1.6	1.7	1.8	2	16.1
F^−^	0.93	1.05	1.04	0.91	0.34
Mineral^d^ [%]	1.33	1.30	1.28	1.39	3.25
HS^−^/H_2_S [mg/dm^3^]	20.6	40.7	23.5	59.3	960
TOC^e^ [mg/dm^3^]	12.4	4.57	29.4	1.06	54.9
N_org_ [mg/dm^3^]	0.77	5.28	0.18	3.82	29.28
TN^f^ [mg/dm^3^]	18.04	29.4	19.5	40.4	132.1
TP^g^ [mg/dm^3^]	BDL	BDL	BDL	BDL	BDL
SLA^h^	Cretaceous (Turon-Senonian)	Cretaceous (Cenomanian)	Cretaceous (Cenomanian)	Neogene-Cretaceous-Jurassic	Upper Jurassic
	Marls and sandstones	Marls and sandstones	Marls and sandstones	Sandstones	Marls and limestones

^a^Depth of water extraction/sampling depth

^b^Electrical conductivity

^c^Below detection limit (1.8 μg/dm^3^ for PO_4_^3−^; 10 μg/dm^3^ for TP)

^d^Mineralization

^e^Total organic carbon

^f^Total nitrogen

^g^Total phosphorous

^h^Stratigraphy and lithology of aquifer

Waters of Busko 17 and C1 are associated only with the Cenomanian (Upper Cretaceous) aquifer consisting of sands and sandstones. Busko 4B water is extracted from the Cenomanian and Turon-Senonian deposits of sandstones, marly limestones, and aquiclude marls [12, 29]. Water of Dobrowoda is associated mostly with sands of Miocene (Neogene), but probably it is also supplied from the deeper Cenomanian and Kimmeridgian-Tithonian (Upper Jurassic) deposits [18]. Water of Welnin is extracted from Upper Jurassic marls and limestones [17].

Sulfidic waters of Busko-Zdrój area are expected to be poorly renewable or even unrenewable. The Cenomanian aquifer has a fault-block structure and it splits into a few isolated zones with no or little connections. This hinders lateral flow of water and makes it difficult to establish recharging area for the exploited reservoirs [17].

Isotopic composition of Busko (4B, 17, C1) and Dobrowoda sulfidic waters reminds of Holocene waters; however, they are deprived of tritium and radiocarbon which suggests that they originate from interglacial infiltration of meteoric waters, which was probably occurring 75,000 to 140,000 years ago. The exception is the sulfidic water of Welnin, which is older and may have originated from precipitation infiltration in the warm Pre-Pleistocene climates [17].

### Physical and Chemical Analyses

Basic water parameters (temperature, pH, Eh, and electrical conductivity) were field-measured with CPC-401 pH/Eh/conductivity-meter (Elmetron, Zabrze, Poland). Physicochemical analyses were performed by the accredited laboratory as part of regular monitoring. Concentrations of inorganic compounds (cations, anions, and H_2_S/HS^−^) were determined according to Polish norms of water quality testing, using the following methods: spectrophotometric (for Fe^2+^, NH_4_^+^), gravimetric and titration (for Ca^2+^, Mg^2+^, SO_4_^2−^, HCO_3_^−^, Cl^−^, Br^−^, I^−^, HS^−^/H_2_S), flame atomic absorption spectrometry (for Na^+^, K^+^), and ion-exchange chromatography (for NO_3_^−^, PO_4_^3−^). Total organic carbon (TOC) was measured with TOC 5000 Analyzer combined with an SSM-5000A module (Shimadzu, Kyoto, Japan). Total nitrogen (TN) and phosphorous (TP) were measured after mineralization, using Nessler or molybdenum blue methods, respectively, followed by UV-VIS spectrophotometry (Spectroquant photometer SQ 118, Merck, Darmstadt, Germany). Organic nitrogen (N_org_) was calculated as the difference between TN and the sum of inorganic N forms. Analyses were performed in triplicate and data given in Table 1 are mean values.

### Total and Live/Dead Cell Counts

The total number and the viability of microbial cells were determined by standard AODC (acridine orange direct count) and L/D (live/dead count) assays, respectively, using the Nikon Eclipse E200 fluorescence microscope (Nikon Instruments, Tokyo, Japan). One-milliliter water samples were filtered through black polycarbonate membranes with 0.22 μm pore size (EMD Millipore, Billerica, MA, USA). In the AODC assay, cells captured on the filter surface were stained for 2 min with acridine orange water solution (100 mg/dm^3^) and rinsed with deionized H_2_O. In the L/D assay, cells captured on the filter surface were stained for 20 min with a mixture of propidium iodide and Syto 9 Green (1:1). Pieces of filters were immediately placed on slides and viewed at a ×1000 magnification. Then, color micrographs were taken using Nikon Eclipse E200 with DS-Fi1 digital microscope camera and the NIS-Elements F3.0 software package (Nikon Instruments, Tokyo, Japan). The number of cells on the micrographs was digitally counted using the image analyzer MultiScan Base v.14 (Computer Scanning Systems Ltd., Warsaw, Poland), and then it was expressed as a number of cells per milliliter (Table 2).

**Table 2:** Microbiological characteristics of waters: total cell counts, cell viability, and 16S rDNA copies per 1 ng of environmental DNA

	Busko 4B	Busko 17	Busko C1	Dobrowoda	Wełnin
TNC^a^ [cells/cm^3^]	6.5 ± 3.2E + 5	5.0 ± 1.3E + 5	2.3 ± 0.8E + 5	2.6 ± 1.5E + 5	3.4 ± 0.8E + 5
Viable cells [%]	14.0	11.5	54.9	62.8	39.5
Bacterial 16S rDNA copies	4.90 ± 0.34E + 7	1.71 ± 0.66E + 7	5.19 ± 0.10E + 7	3.56 ± 0.91E + 7	1.66 ± 0.12E + 7
Archaeal 16S rDNA copies	7.35 ± 0.36E + 4	3.71 ± 0.64E + 4	1.64 ± 0.18E + 5	3.81 ± 0.52E + 4	3.63 ± 0.13E + 4
% of archaeal 16S rDNA	0.15	0.22	0.32	0.11	0.22

^a^Total number of cells

### Extraction of Total Community DNA

Water samples (250 ml) were filtered through sterile polycarbonate membranes with 0.22 μm pore size (EMD Millipore, Billerica, MA, USA). Filters were fragmented into pieces, put into tubes, and immediately used for total community (total genomic) DNA isolation with the method developed by Zhou and coworkers [30]. DNA concentration was measured using Qubit 2.0 (Invitrogen, Carlsbad, CA, USA) and Qubit dsDNA HS Assay Kit (Thermo Fisher Scientific, Waltham, MA, USA).

### Sequencing Experiment Design

High-throughput sequencing of bacterial and archaeal 16S rDNA amplicons was performed on Illumina MiSeq (Illumina, San Diego, CA, USA). The design of the experiment was based on the amplicon preparation by two rounds of PCR during which MIDs (multiplex identifiers for sample barcoding) and sequencing adapters (P5 or P7) were added to the generated products in such a way that it enabled their binding to the Illumina MiSeq flow cell.

We used 357F [31] and 786R [32] primers for bacterial amplicons, while for archaeal ones, due to the lack of a suitable pair giving a specific product of the desired length, we designed a pair consisting of 513F: GGT GYC AGC CGC CGC GGT AA or 915R: GTG CTC CCC CGC CAA TTY CT. The pairs’ specificity was tested with PCR using Genomic DNA from Microbial Mock Community A, HM-278D (reference material for Human Microbiome Project; BEI Resources, VA, Manassas, USA).

The 3′ ends of the first-round PCR primers consisted of universal sequences targeted at 16S rRNA genes (357F: CCT ACG GGA GGC AGC AG or 786R: ACC AGG GTA TCT AAW CC for the bacterial community [31, 32] and 513F: GGT GYC AGC CGC CGC GGT AA or 915R: GTG CTC CCC CGC CAA TTY CT for the archaeal community, this paper). These parts were preceded by 5′ overhangs consisting of M13 sequences (GTT TTC CCA GTC ACG AC for forward primers and CAG GAA ACA GCT ATG AC for reverse ones). In the second round of PCR, M13 sequences served as priming sites for other primers containing the 3′ end: M13 sequences, unique 9-mer MIDs (multiplex identifiers) and P5 (AAT GAT ACG GCG ACC ACC GAG ATC TAC AC) or P7 (CAA GCA GAA GAC GGC ATA CGA GAT) adapters to the flow cell of the Illumina MiSeq sequencer (Table 3).

**Table 3:** Characteristics of developed primers for 16S rDNA amplicon sequencing

Target	Primer	Sequence	*T*_A_ (°C)
Bacteria	M13-357F	GTT TTC CCA GTC ACG ACC CTA CGG GAG GCA GCA G	55
	M13-786R	CAG GAA ACA GCT ATG ACC GTC GTC GTC GGA CCA GGG TAT CTA AWC C	55
	786R_i5	GGW TTA GAT ACC CTG GTC CGA CGA CGA CGG TCA TAG CTG TTT CCT G	–
Archaea	M13-513F	GTT TTC CCA GTC ACG ACG GTG YCA GCC GCC GCG GTA A	62.5
	M13-915R	CAG GAA ACA GCT ATG ACG TGC TCC CCC GCC AA	62.5
	915R_i5	AGR AAT TGG CGG GGG AGC ACG TCA TAG CTG TTT CCT G	–
U_SR-PCR_	P5-M13F	*AAT GAT ACG GCG ACC ACC GAG ATC TAC AC***X**_**9**_ GTT TTC CCA GTC ACG AC	54
	P7-M13R	*CAA GCA GAA GAC GGC ATA CGA GAT***X**_**9**_ CAG GAA ACA GCT ATG AC	54

Underlined sequences are complementary to 16S rDNA genes; M13-357F and M13-513F served as first-round forward and sequencing read 1 primers; M13-786R and M13-915R served as first-round reverse and sequencing read 2 primers; U_SR-PCR_ universal second-round PCR primers; Illumina flow cell adapters are italicized; **X**_**9**_ 9-mer multiple identifier

*T*_*A*_ annealing temperature

High-quality (HPLC-purified) first-round PCR primers, forward and reverse ones, served also as custom sequencing primers for read 1 and read 2, respectively. Additionally, custom i5 index read primers, being the reverse compliment sequences of read 1 primers, were used during sequencing (Table 3).

Both published (357F, 786R) and newly designed archaeal (513F, 915R) 16S rRNA primers were evaluated against the newest version of SILVA SSU database (r126 at the time of writing) to establish their specificity and the coverage of sequences [33]. Online TestPrime tool (http://www.arb-silva.de/search/testprime/) was run with settings allowing two mismatches but requiring absolute conservation of the last two nucleotides.

### Preparation of 16S rDNA Amplicon Libraries

Bacterial and archaeal 16S rDNA amplicons (about 500 and 400 bp long, respectively) were generated using 0.5 ng of total community DNA as a template in PCR reactions containing 0.2 mM dNTPs, 0.25 μM first-round primers, 1 U High Fidelity Phusion Polymerase, and the appropriate 1× buffer with 1.5 mM MgCl_2_ (Thermo Fisher Scientific, Waltham, MA, USA). The following cycling conditions were applied: initial denaturation at 98 °C for 30 s; 30 cycles of − 98 °C for 10 s, 55 °C (bacterial primers)/62.5 °C (archaeal primers) for 15 s, 72 °C for 10 s; and final elongation at 72 °C for 5 min. For each water sample, PCR reactions were performed in triplicate (technical replicas to minimize PCR bias). Negative control (containing water instead of environmental DNA) was prepared and amplified along with the studied samples to exclude amplicons coming from contamination of reagents [34]. The concentrations of 16S rDNA amplicons were measured using Qubit dsDNA HS Assay Kit (Thermo Fisher Scientific, Waltham, MA, USA). Then, the equal quantity (50 pg) of each amplicon was used for a second round of PCR with primers carrying unique MIDs and the flow cell adapters. The reaction mixture contained 0.2 mM dNTPs, 0.25 μM second-round primers, 1 U Taq Polymerase, and the appropriate 1× buffer with 1.5 mM MgCl_2_ (Thermo Fisher Scientific, Waltham, MA, USA). The thermal profile of cycling was as follows: initial denaturation at 95 °C for 3 min; 15 cycles of − 95 °C for 30 s, 54 °C for 20 s, 72 °C for 30 s; and final elongation at 72 °C for 5 min. The concentrations of PCR products were measured as mentioned before.

Separate bacterial and archaeal libraries were created by mixing equal quantities of 16S rDNA amplicons, and an additional library contained amplicons obtained from the negative control. Libraries were double purified with Agencourt AMPure XP (Beckman Coulter, Brea, CA, USA), then evaluated using Agilent 2100 Bioanalyzer and High Sensitivity DNA Analysis Kit (Agilent Technologies, Waldbronn, Germany).

### Quantification and Sequencing of Libraries

16S rDNA libraries were quantified with KAPA DNA Library Quantification Kit (KAPA Biosystems, Wilmington, MA, USA). Reactions prepared according to the manufacturer’s protocol were performed on LightCycler 480 System (Roche Applied Science, Penzberg, Upper Bavaria, Germany). The libraries were mixed in equimolar ratio and diluted to obtain one 6 pM sequencing library with 10% PhiX Control DNA (Illumina, San Diego, CA, USA). Sequencing was performed on Illumina MiSeq using MiSeq Reagent Kit v3 (600 cycles) (Illumina, San Diego, CA, USA). HPLC-purified custom sequencing primers were mixed with Illumina ones. Demultiplexing of indexed reads was made according to Illumina standard protocol.

### Real-Time Quantitative PCR

Absolute quantification of the bacterial and archaeal 16S rRNA gene copies in total community DNA was done with quantitative PCR (qPCR). Reactions containing 1× LightCycler 480 SYBR Green I Master (Roche Applied Science, Penzberg, Upper Bavaria, Germany), 500 nM primers, and DNA (10 pg) were performed on the LightCycler 480 System (Roche Applied Science, Penzberg, Upper Bavaria, Germany). Primers for amplification were 357F and 786R for Bacteria or 513F and 915R for Archaea (see “Sequencing Experiment Design” section). Each DNA sample was analyzed in triplicate. The cycling conditions included initial denaturation at 95 °C for 10 min; 40 cycles of − 95 °C for 10 s, 56 °C (bacterial primers)/54 °C (archaeal primers) for 15 s, and 72 °C for 20 s. The melting curve step consisted of denaturation at 95 °C for 5 s, annealing at 65 °C for 1 min, and continuous temperature rise to 95 °C.

A fragment of bacterial or archaeal 16S rDNA sequence, cloned into pCR4 TOPO vector (Thermo Fisher Scientific, Waltham, MA, USA), was used as control DNA for standard curve preparation. Tenfold serial dilutions of plasmids contained 2.9 × 10^3^ to 2.9 × 10^8^ copies of bacterial or 3.9 × 10^3^ to 3.9 × 10^8^ copies of archaeal 16S rDNA per reaction. Negative controls were analyzed parallel to the samples under study.

The results of qPCR were expressed as copies of bacterial and archaeal 16S rRNA genes in 1 ng of environmental DNA. The ratio of archaeal to bacterial genes was calculated as follows: copies of archaeal genes in 1 ng/sum of bacterial and archaeal gene copies in 1 ng * 100%.

### Bioinformatics Analyses

Quality trimming of FastQ files was performed with Sickle [35] and then reads were corrected with BayesHammer [36]. Paired-end reads were generated with Pandaseq [37] and the resulting contigs (hereafter referred to as the “sequences”) were analyzed with Mothur v1.35 [38]. Sequences containing degenerated bases or homopolymers longer than 8 nts were removed (screen.seqs) and the remaining ones were classified with a naive Bayesian classifier using SILVA v.126 reference database (classify.seqs) [33, 39]. Bacterial and archaeal sequences were divided (get.lineage) and then analyzed separately as described earlier [40]. Briefly, the sequences were dereplicated (unique.seqs) and aligned to SILVA v.126 template alignment. Unaligned reads were removed and the alignment was trimmed to positions immediately following the sequencing primers. Putative chimeric sequences were identified with UCHIME (chimera.uchime) and Perseus (chimera.perseus) and were removed [41, 42]. Operational taxonomic units (OTUs) were constructed via average neighbor (UPGMA) clustering at the 0.03 dissimilarity level. Singletons and doubletons were discarded. The remaining OTUs were assigned taxonomic classification down to the genus level (classify.otu). Rarefaction curves were calculated for the obtained OTUs (rarefaction.single).

### Species Richness and Diversity Analyses

Species richness (OTU observed, Chao1, ACE), Shannon’s diversity (*H′*) and evenness (E) indices were calculated at the 0.03 dissimilarity level with Mothur (summary.single) for thousandfold randomized subsamples of 4000 sequences/sample for Bacteria and 500 sequences/sample for Archaea. Venn diagrams based on the tables of shared OTUs were prepared with Venn Diagrams tool (http://bioinformatics.psb.ugent.be/software/details/ Venn-Diagrams). OTUs with fewer than 5 sequences were discarded to reduce the sequencing errors and the background noise. Heatmaps of microbial communities based on Bray-Curtis and Morisita-Horn distances were generated in R v.3.3.1 (Phyloseq package) using the normalized OTU tables at 0.03 dissimilarity level [43]. Ordination-based heatmaps for the most abundant bacterial and archaeal OTUs were calculated in R (Phyloseq package) using the nonmetric multidimensional scaling (NMDS) method and Bray-Curtis distance [44]. Data given in Table 4 are mean values calculated for each sample consisting of three subsamples, which were sequenced and analyzed separately.

**Table 4:** Sequencing results and diversity analyses

Parameter	MG^a^	Sample				
		Busko 4B	Busko 17	Busko C1	Dobrowoda	Wełnin
Sequencing results^b^						
Raw paired-end reads	B	169,376	63,173	105,277	52,268	44,704
	A	22,886	11,744	8570	6483	9981
HQ nonchimeric paired-end reads	B	65,275	24,414	77,530	29,970	15,968
	A	14,108	7283	5405	4427	6273
OTU construction						
Total no. of OTUs (≥ 5 seqs)	B	280	190	256	226	96
	A	127	115	116	91	137
Species richness^c^						
OTU observed	B	178.9	156.2	170.7	189.5	84.4
	A	66.7	68.1	71.9	60.3	85.0
Chao1	B	305.1	184.5	264.5	216.2	93.4
	A	89.0	81.6	86.0	68.4	103.1
ACE	B	408.1	181.5	332.1	214.8	90.2
	A	91.1	84.0	87.1	68.9	105.5
Diversity indices^c^						
Shannon’s diversity (*H′*)	B	1.87	2.41	2.44	3.11	2.09
	A	3.16	3.39	3.35	3.21	3.66
Shannon’s evenness (*E*)	B	0.36	0.48	0.48	0.59	0.47
	A	0.76	0.82	0.80	0.79	0.83

^a^Microbial group (Bacteria or Archaea)

^b^Values given as a sum of three independently sequenced subsamples

^c^Average values of estimates for thousandfold randomized subsets of 4000 sequences for Bacteria and 500 sequences for Archaea

### Statistical Analyses

Normality of data distribution and homogeneity of variance were checked with Shapiro-Wilk test and Levene test, respectively. The differences between values of species richness estimators were tested with one-way ANOVA with Bonferroni correction, and *p* value lower than 0.05 was considered statically significant. The pairwise differences were checked with Tukey HSD test.

Multivariate analyses were performed with R v.3.3.1 using FactoMineR and Vegan packages [43, 45]. Principal component analysis (PCA) was done to reveal physical and chemical factors influencing the water samples and to show the percentage of variation that could be explained by environmental data. NMDS was performed using the table of normalized OTU counts, Bray-Curtis distance matrix, and 1000 iterations. Envfit function was used to link microbial community composition (OTUs at the 0.03 level) to the environmental variables. Ten environmental parameters were selected for both PCA and NMDS analyses: Eh, temperature, depth, and concentrations of Na^+^, Cl^−^, F^−^, SO_4_^2−^, H_2_S, TOC, and TN. pH was excluded since its values were close to neutral in all samples. All data were normalized to zero mean and unit variance. Correlation matrix of environmental variables was calculated prior to PCA and NMDS. Among the parameters showing strong positive correlation (> 0.95), the only one variable was selected and it was plotted as a vector representing all of them (this step reduced the number of dimensions in the ordination; full analyses are available in Supplementary data, Fig. S8–9). The significance of environmental variable correlation with community data was tested with Monte Carlo permutation test applying 10,000 permutations. Bonferroni-corrected *p* values lower than 0.05 were considered significant.

## Results

### Sulfidic Water Geochemistry

All the analyzed sulfide waters were Cl-Na, H_2_S, I, and F type with predomination of sodium and chloride; however, other ions, like Ca^2+^, Mg^2+^, and SO_4_^2−^, were also common. Waters differed significantly in terms of physicochemical parameters, particularly temperature, salinity, and concentrations of H_2_S, TOC, and TN/N_org_ (Table 1). Water of Welnin was 3.2% brine with extremely high H_2_S content (near 1 g/dm^3^) and the highest organic carbon and nitrogen (54.9 and 132.1 mg/dm^3^, respectively). Nitrate was the major form of N in Welnin (63.2 mg/dm^3^). Other waters had mineralization of 1.3–1.4%, H_2_S concentration in the range of 20.5 to 59.3 mg/dm^3^, and they were poorer in organic carbon (1.6–29.4 mg/dm^3^) and nitrogen (18.04–40.4 mg/dm^3^). Ammonium was the major form of N, while nitrate content was low (0.55–0.63 mg/dm^3^). pH in all collected samples was close to neutral (from 6.6 to 7.8), the redox potential was negative, and the temperature varied between 12.4 and 22.8 °C, depending on the depth of extraction.

The principal component analysis performed on the physicochemical data showed dispersion of the sampling sites (Supplementary Fig. S3). The first and second PCA axes taken together explained 84.9% of the observed variation (61.8 and 23.1%, respectively). Some environmental parameters showing strong positive correlation were represented by one variable. Out of five variables, four were statistically significant and they are given in order of increasing *p* value: H_2_S (correlating with Eh, salinity, and TN), F, temperature (correlating with depth), and TOC. Three water samples (4B, 17, and Dobrowoda) were similar to each other and they grouped together on the PCA plot. Two remaining samples were distantly located and they might be strongly influenced by two different variables. In case of Busko C1, temperature and depth (corresponding to pressure) seemed to be essential parameters. Water of Welnin was the most distinct sample and it was characterized by the elevated salinity and higher content of H_2_S, TOC, and TN/N_org_.

### Total Cell Counts and 16S rDNA Quantification

The total numbers of cells in the analyzed samples of sulfidic water were similar and ranged from 2.3 to 6.5 × 10^5^ cells/cm^3^ (Table 2). The highest number of cells was detected in the sample Busko 4B, while three times lower value was noticed in the water from Busko C1. However, the differences were not statistically significant (*p* > 0.05). The morphological diversity of microorganisms was very low. Mainly short and long rods were recognized among the observed shapes (Supplementary Fig. S2). The viability of cells in the samples varied from 11 to 62%.

Evaluation of bacterial 16S rRNA gene copies showed little variation across the samples and ranged from 1.66 to 5.19 × 10^7^ per 1 ng of total community DNA (Table 2). The numbers of archaeal 16S rRNA gene copies, reaching from 3.63 × 10^4^ to 1.64 × 10^5^, were almost three orders of magnitude lower than bacterial ones. The archaeal sequences constituted much less than 0.5% of the total estimated 16S rRNA gene copies (from 0.11% in Dobrowoda to 0.32% in Busko C1).

### Evaluation of Primers

The coverage of bacterial 16S rDNA sequences in the SILVA v.126 database was 77.1% with no mismatches allowed. When two mismatches were accepted, the coverage raised to 94.8% for Bacteria, but the possible amplification of 67.4% of archaeal sequences was also predicted.

The universal primers targeted at Archaea covered 79.7% of archaeal sequences with no mismatches allowed and 90.6% with two mismatches accepted. No hits for Bacteria were detected. Archaeal primers tested in PCR using bacterial mock community as a template gave no amplicons.

### Sequencing Results

In total, 494,462 raw paired-end reads were obtained from sequencing. Bioinformatics data processing (chimeras, singletons, and doubletons removing) resulted in 250,653 (50.7% of the total number) high-quality nonchimeric reads, out of which 213,157 (43.1%) were assigned to the bacterial 16S rDNA sequences and 37,496 (7.6%) to the archaeal ones (Table 4). The median lengths of bacterial and archaeal reads aligned to the SILVA v.126 template alignment were 427 and 386 nts, respectively.

Reads obtained from negative controls were removed from the dataset (details and the list of “contaminating” genera are given in the Supplementary Material). Sequencing data were deposited in NCBI SRA under accession number SRP097822.

### Composition of Microbial Communities

**Fig. 1:**
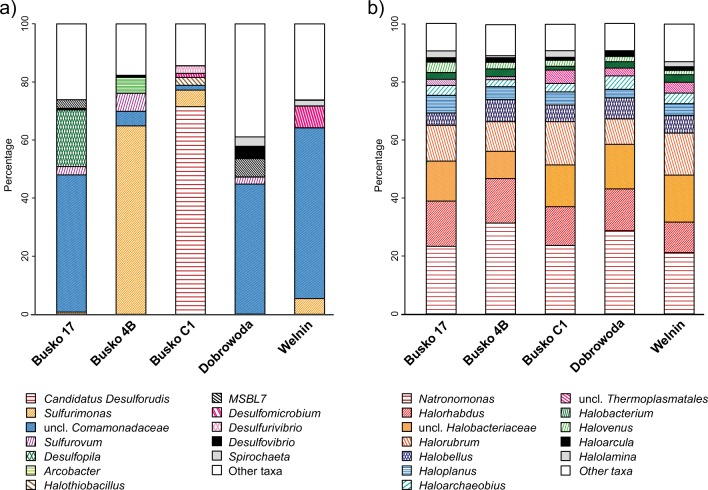
**a**, **b** Percentage composition of microbial communities

High-quality 16S rDNA sequences (213,157) were assigned to *Bacteria*. Sequences affiliated with *Proteobacteria* and *Firmicutes* were the most abundant ones (65.4 and 27.8% of total reads, respectively). *Proteobacteria* predominated in the libraries derived from Dobrowoda, Busko 17, Welnin, and Busko 4B (77.4 to 94.4%, Supplementary Table S3). *Firmicutes* prevailed in the C1 sample, where they reached 73.6%, and were followed by *Proteobacteria* (24.4%). The representatives of other phyla were rarely encountered in the analyzed communities and they belonged mainly to *Bacteroidetes*, *Spirochaete*, and *Nitrospirae* (Fig. 1a). At the class level, Busko 17, Dobrowoda, and Welnin libraries were similar and they differed from Busko 4B one. In the three first samples, *Beta*- (45.5 to 61%) and *Deltaproteobacteria* (14.2 to 27.7%) were the most numerous and they were followed by *Epsilonproteobacteria* (2.9 to 6.4%). Busko 4B was almost completely dominated by *Epsilonproteobacteria* (76.9%), while *Delta*- and *Betaproteobacteria* constituted 7.2 and 5.6%, respectively. In Busko C1, the high representation of *Clostridia* was observed (72.6%), but among *Proteobacteria*, mild domination of delta and epsilon classes was visible (Supplementary Fig. S10). At the family and genus levels, the differences between samples were more distinct. Members of an unclassified genus within *Comamonadaceae* prevailed in Busko 17, Dobrowoda, and Welnin where they constituted 44.4 to 57.8% of communities (Fig. 1a). Moreover, *Desulfopila* (*Desulfobulbaceae*) was found to be common in Busko 17 (19.4%), but it was absent from other samples. In Dobrowoda, the second most numerous genus was *MSBL7* (6%, *Desulfobulbaceae*), while in Welnin, it was *Desulfomicrobium* (7.4%, *Desulfomicrobiaceae*). Busko 4B was abundant in *Sulfurimonas* (64.9%) and *Sulfurovum* (6.2%) of *Helicobacteraceae*. Busko C1 was dominated by *Candidatus Desulforudis* (71.4%, *Peptococcaceae*), that was virtually absent from other libraries (0.01 to 0.28%). Additionally, a small representation of *Sulfurimonas* (5.7%) was also found in Busko C1.

**Fig. 2:**
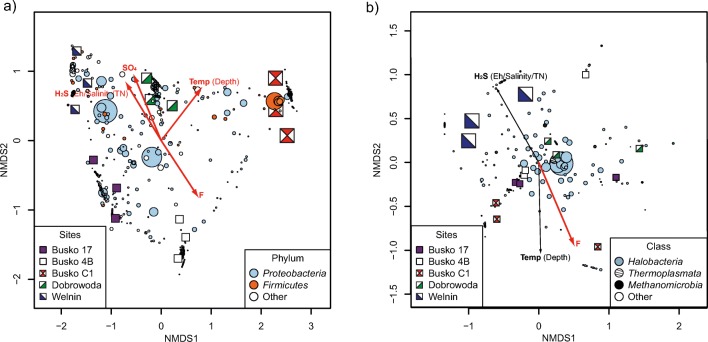
**a**, **b** Bacterial OTUs gathered around specific sites

Archaeal communities were less diverse and more similar to each other than the bacterial ones. Out of 37,496 high-quality sequences, 99.4% were assigned to *Euryarchaeota* (Supplementary Table S3). *Thaumarchaeota* were represented only by 0.3% of sequences and the other 0.3% remained unclassified (Supplementary Fig. S11). In all analyzed libraries, members of the *Halobacteria* class (93.0 to 96.7%) and its family *Halobacteriaceae* (92.1 to 95.8%) dominated (Fig. 2b). *Thermoplasmata* class was also encountered but it was rare (1.7 to 5.6%). At the genus level, the prevalence of *Natronomonas* (21.2 to 31.3%) was observed; however, three other genera were also common: *Halorhabdus* (10.5 to 15.6%), unclassified *Halobacteriaceae* (9.4 to 16.2%), and *Halorubrum* (8.8 to 14.9%) (Fig. 1b). The differences in the composition of archaeal communities were caused by rare genera (with the contribution below 3%). Nevertheless, all detected genera belonged to halophilic archaea.

NMDS ordination plots showed different structures of bacterial and archaeal communities. Many bacterial OTUs were gathered around specific sites (Fig. 2a). The remaining bacterial OTUs were widely dispersed over the area between the site points and only these OTUs were common for more than one sample. Four statistically significant environmental variables could explain the diversification of bacterial communities: H_2_S (correlating with Eh, salinity, and TN), temperature (correlating with depth), F, and SO_4_^2−^. Archaeal NMDS showed that most of the OTUs were grouped in the central part of the plot indicating that similar archaeal communities were found in each sample (Fig. 2b). Only the minor amount of rare archaeal OTUs was gathered around specific sites. Fluoride concentration was the only environmental variable which was statistically significant in the analysis. This result suggests that some unknown factors may influence the composition of archaeal communities.

### Bacterial Groups Potentially Involved in Sulfur Cycling

**Fig. 3:**
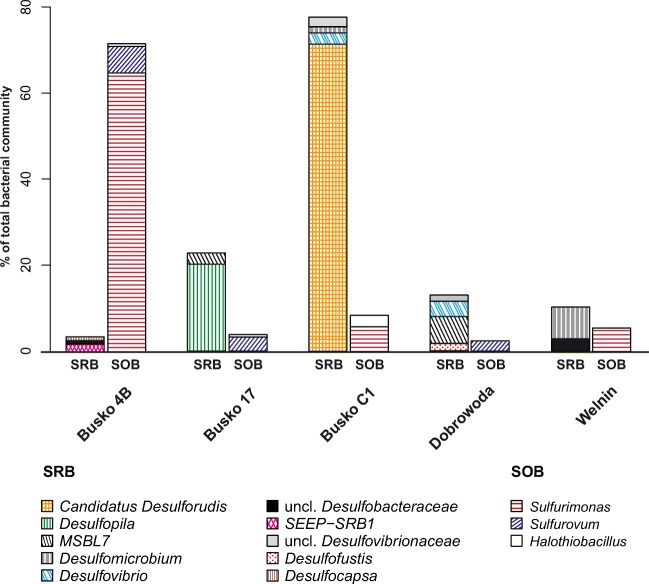
Bacterial groups related to sulfur cycling detected in the analyzed sulfidic waters

Bacterial groups related to sulfur cycling were detected in the analyzed sulfidic waters. SRB were particularly abundant in Busko C1, where they constituted almost 80% of the entire bacterial community (Fig. 3). In other samples, SRB were still abundant and their contribution reached from 6.7 to 27.1%. Taxonomic diversification of SRB was very high and different genera were present in each library. Busko C1 was almost completely dominated by *Candidatus Desulforudis* (71.4%); two other genera observed were *Desulfurivibrio* (2.6%) and unclassified *Desulfovibrionaceae* (2.3%). *Desufopila* (19.4%) prevailed in Busko 17; however, *MSBL7* (2.7%) and *SEEP-SRB2* (0.9%) were also detected. In Dobrowoda, SRB belonged mostly to *MSBL7* (6%), *Desulfovibrio* (3.6%), unclassified *Desulfovibrionaceae* (1.4%), and *Desulfofustis* (1.4%). In Welnin, *Desulfomicrobium* (7.4%) and unclassified *Desulfomicrobiaceae* (2.8%) were found, and they constituted 10% of the total bacterial community. Busko 4B had the smallest representation of SRB among which *Desulfobulbaceae* (2.1%) with *Desulfobulbus* and *Desulfocapsa*, *SEEP-SRB1* (1.8%), *SEEP-SRB2* (0.5%), and unclassified *Desulfobacteraceae* (0.8%) were identified.

Sulfide and SOB were highly abundant in Busko 4B (Fig. 3). The contribution of genera *Sulfurimonas* (64.9%), *Sulfurovum* (6.2%), and *Acidithiobacillus* (0.8%) to the entire bacterial community of Busko 4B was near 72%. In the remaining libraries, SOB were less numerous and they reached from 3.2 to 9.1. *Sulfurimonas* (5.7%) and *Halothiobacillus* (2.7%) were found in Busko C1, while only *Sulfurimonas* (5.5%) was detected in Welnin. Representatives of SOB found in Busko 17 and Dobrowoda were similar and they consisted mainly of *Sulfurovum* (2.9 and 2.4%, respectively), but lower contributions of *Halothiobacillus* (0.6 and 0.4%) and *Sulfurimonas* (0.4% both) were also observed.

**Fig. 4:**
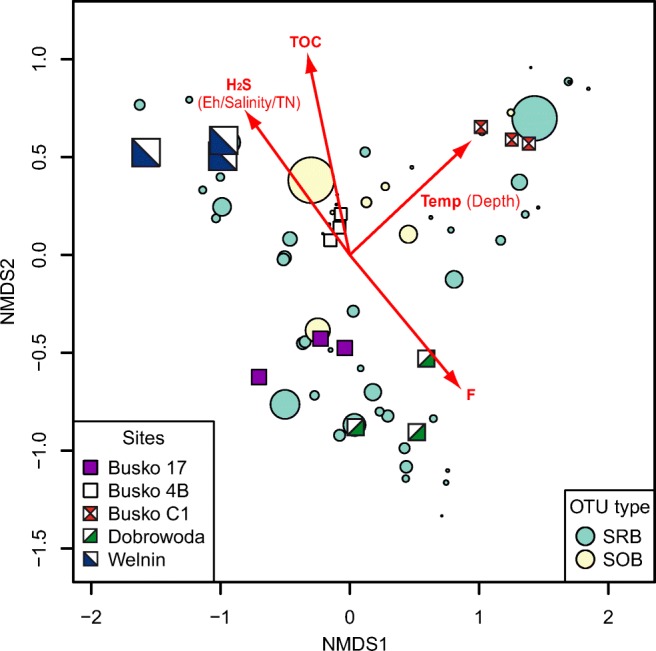
NMDS plot showing dispersion of sampling sites and bacterial genera related to sulfur cycling

NMDS plot showed wide dispersion of sampling sites and bacterial genera related to sulfur cycling (Fig. 4). Four environmental variables given on the ordination plot (H_2_S, TOC, temperature, and F) were statistically significant (*p* > 0.05), and they may be reflected in the unique pattern of SRB and SOB in each sample. *Candidatus Desulforudis*, the most abundant OTU of SRB, was placed close to the site Busko C1, characterized by elevated values of temperature and pressure related to depth of water extraction. *Sulfurimonas*, the most abundant OTU of SOB, was specific to the site Busko 4B, which was the shallowest one but had the increased concentration of TOC.

### Species Richness and Diversity Analyses

OTU numbers, Chao1, ACE, Shannon’s *H′*, and rarefaction curves were calculated at the 0.03 dissimilarity level to estimate the diversity of the bacterial and archaeal communities in the analyzed waters. The total number of bacterial OTUs (containing at least 5 sequences) constructed for the whole dataset was 850. The number of observed bacterial OTUs, found in normalized subsets of 4000 randomly picked sequences, ranged from 84 to 189 (for Welnin and Dobrowoda, respectively) (Table 4). However, nonparametric indices, Chao1 and ACE, predicted that Busko 4B might have the highest total number of OTUs reaching even 400. For three samples (Busko 17, Dobrowoda, and Welnin), the differences between the observed and estimated species richness were rather small, and potentially, 80 to 90% of total diversity was captured by the analysis. Two other samples (Busko 4B and C1) seemed to have higher species richness than it was observed and up to 50% of species was suggested to be not represented. ANOVA with Tukey’s test showed that Welnin with the lowest bacterial species richness differed significantly from the rest of the waters (*p* < 0.01). Rarefaction curve for Welnin was strongly flattened which indicated high coverage of sample diversity (Supplementary Fig. S5a). The curves for Busko 4B and C1 were far from saturation, which was consistent with calculated values of species richness indices. These bacterial communities were moderately sampled and higher number of sequences would be required to increase the observed diversity.

Shannon’s diversity for the bacterial communities was relatively low and ranged from 1.87 to 3.11, while Shannon’s evenness ranged from 0.36 to 0.59 (for Busko 4B and Dobrowoda, respectively), which suggested that a few species dominated the assessed microbiomes. Dobrowoda, with the highest estimated diversity and evenness, differed significantly (*p* < 0.01) from the remaining waters. Venn diagrams showed that communities strongly differed from each other and only 8 out of 850 OTUs (≥ 5 sequences) were found in all the analyzed waters, while most of the remaining OTUs were sample-specific (Supplementary Fig. S4a). Among these 8 OTUs, only OTU002 was abundantly represented in all the samples, and in three of them, its contribution was at least 45%. This OTU belonged to unclassified genus of *Comamonadaceae* (Table 5) that was most similar but not strictly affiliated with *Curvibacter* (96–98% of similarity to different *Curvibacter*-type strains and 96–97% of similarity to *Acidovorax*-type strains)*. Curvibacter* itself accounted for no more than 0.43% of sequences (data not shown). When OTU002 was reanalyzed at the 0.01 dissimilarity level, it showed very high homogeneity since close to 83% of sequences were grouped within one sub-OTU. Other OTUs shared by all samples were rare and their contribution to microbial diversity was extremely small (Supplementary Table S2). Nevertheless, a heatmap of abundance (Supplementary Fig. S6a) showed that each analyzed sample displayed a specific pattern of a few predominating OTUs, that constituted from 50 to 70% of the community (Table 5). Heatmap of distances between bacterial communities (Supplementary Fig. S7a-b) revealed that Busko 17, Welnin, and Dobrowoda were similar to each other and different from the remaining samples.

**Table 5:** The most abundant bacterial and archaeal OTUs in water samples

OTU	Contribution to the microbial diversity (%)					Taxonomical affiliation
	4B	17	C1	D	W	
001	65.4	0	5.7	0.09	5.5	Bacteria; *Proteobacteria*; *Epsilonproteobacteria*; *Campylobacterales*; *Helicobacteraceae*; *Sulfurimonas*
002	5.1	47.9	1.7	45.0	57.8	Bacteria; *Proteobacteria*; *Betaproteobacteria*; *Burkholderiales*; *Comamonadaceae*; unclassified genus
003	0.02	0.09	49.3	0	0.01	Bacteria; *Fimicutes*; *Clostridia*; *Clostridiales*; *Peptococcaceae*; *Candidatus Desulforudis*
004	0	0.03	9.3	0	0	Bacteria; *Fimicutes*; *Clostridia*; *Clostridiales*; *Peptococcaceae*; *Candidatus Desulforudis*
005	0	19.9	0	0	0	Bacteria; *Proteobacteria*; *Deltaproteobacteria*; *Desulfobacterales*; *Desulfobulbaceae*; *Desulfopila*
017	0	0	0	5.0	0	Bacteria; *Proteobacteria*; *Deltaproteobacteria*; *Desulfobacterales*; *Desulfobulbaceae*; *MSBL7*
001	29.5	22.0	21.4	26.4	19.6	Archaea; *Euryarchaeota*; *Halobacteria*; *Halobacteriales*; *Halobacteriaceae*; *Natronomonas*
002	9.7	11.8	12.2	7.7	10.5	Archaea; *Euryarchaeota*; *Halobacteria*; *Halobacteriales*; *Halobacteriaceae*; *Halorubrum*
003	6.3	2.5	4.2	4.9	3.5	Archaea; *Euryarchaeota*; *Halobacteria*; *Halobacteriales*; *Halobacteriaceae*; *Halobellus*
004	5.6	4.2	3.9	2.6	2.6	Archaea; *Euryarchaeota*; *Halobacteria*; *Halobacteriales*; *Halobacteriaceae*; *Halorhabdus*
005	2.8	2.3	1.9	4.7	1.7	Archaea; *Euryarchaeota*; *Halobacteria*; *Halobacteriales*; *Halobacteriaceae*; *Halorhabdus*

Values of estimators computed for the archaeal communities differed from bacterial ones. The total number of OTUs (≥ 5 sequences) constructed for the dataset was 372. The highest average number of observed OTUs in the normalized subset of 500 sequences was 85 and it was found in Welnin. Nonparametric indices values predicted that OTU numbers in Welnin reach 105. In the remaining samples, the number of OTUs was lower and ranged from 60 to 72; however, the differences were not statistically significant. Chao1 and ACE estimators compared to the number of observed OTUs suggested that 70 to 85% of archaeal species were captured by the analyses. Rarefaction curves, except for Busko 4B, were flattened, which indicated the high level of sampling of the remaining waters (Supplementary Fig. S5b). The archaeal diversity and evenness indices (reaching 3.16–3.66 and 0.76–0.83, respectively) were higher than bacterial ones and the communities were less dominated. The Venn diagram showed that 24 OTUs were shared by all assessed communities (Supplementary Fig. S4b). Two of these OTUs (OTU001 and OTU002) were abundantly represented in all water samples (23.8 and 10.4% in average, respectively). They belonged to genera *Natronomonas* and *Halorubrum* (Table 5). Heatmaps of both OTU’s abundance and archaeal community distances (Supplementary Fig. S6b, Fig. S7c-d) were consistent with other analyses and showed low variation (high similarity) across the water samples.

## Discussion

In this study, we analyzed the composition and species richness of bacterial and archaeal communities of natural sulfide-rich subsurface waters extracted within and in the vicinity of Busko-Zdrój town (Carpathian Foredeep, south-eastern Poland). In spite of the relative proximity of the collection sites (from 350 m to 19 km), water samples differed from each other in specific physicochemical parameters: temperature related to the depth of extraction, salinity, content of H_2_S, TOC, and TN/N_org_. Physicochemical data, supported by hydrogeological investigations by Zuber et al., suggest separation of the reservoirs, from which the water samples were collected. The lack of hydrological connection results from the fault-block structure of the region, which hinders lateral flow of water between reservoirs [17, 18, 29]. Our studies revealed microbial life in the subsurface sulfide-rich waters of the Carpathian Foredeep. Interestingly, microbial communities existing under different sulfide concentrations varied in species richness but neither in the Shannon’s diversity (*H′* values were strongly influenced by the domination of some specific taxa) nor the cell number. It seems that extremely high concentration of HS^−^/H_2_S in combination with elevated salinity affected the species richness (significantly decreased the number of bacterial OTUs); however, the impact of these abiotic factors might be modified with raised levels of TOC and TN in such a way that the diversity of the whole community was not dramatically changed. Direct counts showed that the total number of cells in all analyzed sulfide-rich waters was similar irrespectively of H_2_S and nutrient content and ranged from 2.3 to 6.5 × 10^5^ cells/cm^3^. These values are typical of many subsurface waters extracted from the depths down to 1000 m [3, 9, 46, 47]. Density of cells in the waters of Carpathian Foredeep was slightly higher compared to the only one described sulfide-rich water of the Ust’-Katcha Resort Spring (240 mg H_2_S/dm^3^), Russia [22]. Pimenov et al. reported that the number of microbial cells in water extracted from the depth of 204–501 m bgl was 4–5 × 10^4^ cells/cm^3^; however, this water contained elevated salt concentration (65 to 80 g/dm^3^). Quantification of microbial 16S rRNA gene copies revealed that the communities of sulfidic waters are dominated by bacterial inhabitants while archaeal ones are a minority. This observation is in line with the results of similar analyses done for deep fracture fluids [46, 47] in which *Archaea* were found to constitute only the small portion of the communities.

The result of the community structure analysis strongly depends on the primer sets chosen for amplification of 16S rDNA fragments. Both bacterial and archaeal pairs of primers used in this study performed well in the evaluation test against the SILVA database, showing high specificity and coverage of 16S rRNA genes. This relatively low bias, typical for every PCR-based community analysis, supports the validity of the demonstrated results; however, it should be noted that some taxa still might be underrepresented.

Differences in physicochemical parameters of waters under study seemed to be reflected mostly in the bacterial community structures, since the archaeal ones were similar in all the analyzed samples. Bacterial communities strongly differed between samples. Out of 850 bacterial OTUs, only 8 were shared by all samples, but only one unclassified betaproteobacterial OTU (similar to *Curvibacter* within *Comamonadaceae*), with its abundance ranging from 1.7 to 57.8% of total community, may be considered as the potential “core OTU” of sulfide-rich waters. The remaining OTUs, with the abundance below 1% and uneven distribution across the samples, belonged rather to the “rare biosphere.” The *Comamonadaceae*-affiliated OTU was observed in the waters collected from the reservoirs having no hydraulic connection at the present time. Surprisingly, this specific OTU showed high homogeneity even at the 0.01 dissimilarity level. We suspect that it may have originated from an ancient bacterial community inhabiting the water of primary Cenomanian aquifer before orogenic processes of the Laramide phase caused the development of aquifer’s fault-block structure and separation of its parts. Upward and downward translocations of the aquifer’s parts probably resulted in significant changes of environmental conditions (e.g., depth, temperature, and pressure and rocks in contact with water) affecting the composition of microbial communities. The results obtained in this study suggest that the *Comamonadaceae* may possess wide adaptation potential. According to our observation, the abundance of the unclassified *Comamonadaceae* similar to *Curvibacter* was increased in the samples extracted from depths of 148 to 300 m bgl and having elevated concentration of H_2_S and nitrogen. We suspect that the appropriate combination of these specific environmental factors may influence the high contribution of *Comamonadaceae* to the groundwater community. Purkamo et al. reported the domination of unclassified *Comamonadceae* in the subsurface waters of Outokumpu Deep Drill Hole (eastern Finland), while Rempfert et al. found that *Comamonadaceae* were ubiquitous in the fluids of the Samail Ophiolite (Sultanate of Oman) [47, 48]. However, Purkamo et al. found that *Comamonadaceae* were abundant (~ 70% of the community) only in waters extracted from 180 and 500 m bgl and their numbers dramatically decreased with depth. The authors assigned *Curvibacter* as well as other representatives of *Comamonadaceae* (*Burkholderiales*) to the “keystone species” of deep fracture fluid communities. These metabolically flexible bacteria are expected to be involved in hydrogen oxidation (if only trace amounts of oxygen, e.g., coming from water radiolysis [49], are available), denitrification, ferric iron reduction, and primary production in oligotrophic subsurface environments [47, 48, 50, 51]. Thus, taking into account the results obtained by other groups, we hypothesized that the members of *Comamonadaceae* are likely to represent archetypal microbial species in sulfide-rich waters of the Carpathian Foredeep.

In three samples of sulfidic waters, where *Comamonadaceae* were strongly dominating, we detected also common co-occurrence of different deltaproteobacterial genera (i.e., *Desulfopila*, *Desulfomicrobium*, *MSBL7*, *Desulfovibrio*, or *Desulfovibrio*-related bacteria) constituting 14 to nearly 28% of the communities. Our observation is similar to the results of Itävaara et al. and Purkamo et al. describing deep water microbial communities consisting mostly of *Comamonadaceae* and minor representation of *Deltaproteobacteria*, among which two genera (*Desulfomicrobium* and *Desulfovibrio*) were shared with sulfidic waters [3, 47]. We conclude that in the aquifers located at smaller depths, beta- and deltaproteobacterial taxa may commonly co-occur. A large body of evidence suggests that Deltaproteobacteria are widespread in subsurface aquatic environments, e.g., in Fennoscandian crystalline bedrock fluids [47, 52], karst groundwater [53, 54], or water associated with Upper Jurassic calcareous formation [55]. *Deltaproteobacteria* are sulfate reducers that use oxidized sulfur compounds as terminal electron acceptors in the process of organic matter degradation under oxygen-depleted conditions. As major players in the S and C cycles, these microorganisms are essential for deep ecosystem functioning [56].

Surprisingly, in the sulfide-rich water, extracted from the 60-m-deep borehole Busko 4B, representations of *Beta*- and *Deltaproteobacteria* were small (taken together, they did not exceed 13%), while epsilonproteobacterial genera *Sulfurimonas*, *Sulfurovum*, and *Arcobacter* were highly abundant (nearly 77%). This dramatic change in the ratios between different proteobacterial classes correlated with low depth and low nitrogen content. Nevertheless, it cannot be excluded that suboxic conditions, which might occur in the shallowest borehole water, contributed to the abundance of *Epsilonproteobacteria*. Macalady et al. indicated that high sulfide to oxygen ratio (> 150) promoted the intensive growth of *Epsilonproteobacteria* (e.g., *Sulfurovum* and *Arcobacter* among the others) in the sulfidic water of the Frasassi cave [57]. We suspect that similar environmental conditions are likely to occur in the shallowest borehole water. The genera *Sulfurimonas*, *Sulfurovum*, and *Arcobacter* detected in this study comprise chemolithoautotrophs, which are ubiquitous in the deep marine waters, e.g., hydrothermal vent fluids and sediments [58]; however, they are also encountered in the intraterrestrial aquifers, e.g., Fennoscandian fracture fluids [52]. Bomberg et al. reported that *Sulfurimonas* strongly dominated (64–95%) in the active community of the sulfidic groundwater found at the depth of 296–347 m bgl. *Epsilonproteobacteria* as sulfur oxidizers are expected to be enriched in the habitats containing high H_2_S content. They may fix CO_2_ using sulfide (*Arcobacter*), elemental sulfur, and thiosulfate (*Sulfurimonas*, *Sulfurovum*) as electron donors [59], thus playing an important ecological role in sulfur cycling.

Bacterial community of the Busko C1 water was strikingly different from the others. At the depth of 660 m bgl, *Firmicutes* constituted 73%, while the share of Proteobacteria decreased to only 24%. *Firmicutes* were almost entirely represented by the genus *Candidatus Desulforudis* (*Clostridia*) consisting of extremely versatile sulfur-reducing bacteria. Chivian et al. reported that *C. Desulforudis* was capable of forming single-species ecosystem, thus living independently of other microorganisms in the 2.8-km-deep fracture water of South African mine [60]. *C. Desulforudis* was also found as a minor member of SRB in Fennoscandian fracture fluids at the depth of 180 to 2260 m bgl; however, the highest representation of this genus was detected below 1820 m [47]. Interestingly, in our study, *C. Desulforudis* was abundant at the depth of 660 m and its high representation seemed to be related to the elevated temperature/pressure and lower nitrogen concentration (Fig. 3a). Bacteria belonging to this genus were shown to possess a wide repertoire of genes encoding versatile metabolic pathways; hence, they may thrive in nutrient-poor habitats [60].

Our results showed that sulfidic waters under study were commonly inhabited by bacteria related to sulfur cycling; however, the ratio of SRB to SOB (as well as co-occurrence of specific genera) differed between samples. In the deepest borehole water, intensive production of S^0^ and H_2_S may be expected due to overwhelming representation of SRB (above 79% of the total community) and small proportion of SOB (9%). On the contrary, in the shallowest borehole water, efficient generation of SO_4_^2−^ may be anticipated, since SOB completely dominated over SRB (73 vs. 7%). In the remaining waters, SRB were more abundant than typical SOB that constituted the minority (3–5%). However, it is also likely that the dominant *Comamonadaceae*, as nitrate reducers [61], potentially contribute to sulfur oxidation (e.g., via denitrification or dissimilatory nitrate reduction pathway coupled to S cycle under anoxic conditions). He et al. identified genes of nitrogen and sulfur metabolism in the genomes of *Comamonadaceae* [62]. Furthermore, analysis of known sulfur metabolism pathways in the KEGG database (Kyoto Encyclopaedia of Genes and Genomes, [63]) revealed that genomes of many members of *Comamonadaceae* harbor the Sox system for thiosulfate oxidation. This further supports our presumption that *Comamonadaceae* play a prominent role in nitrogen and sulfur cycling in these waters.

Unexpectedly, archaeal communities (unlike bacterial ones) displayed low diversity and similar structure in all studied sulfidic waters of Carpathian Foredeep. An analogous observation was made by Purkamo et al. [47], who compared bacterial and archaeal communities of deep Fennoscandian fracture fluids. However, the authors suggested that the results might be affected by low coverage of archaeal sequences by PCR primers. We cannot exclude the PCR bias, but it is also possible that the structure of archaeal community was maintained due to wide adaptation potential of its members.

Archaeal communities revealed in this work almost entirely consisted of *Halobacteriaceae* (phylum *Euryarchaeota*). Representations of unclassified *Thermoplasmatales* and *Deep Sea Hydrothermal Vent Group 6* (*DHVEG-6*) were marginal. Surprising abundance of halophilic *Archaea* was also described in a low-salt, sulfidic lake found in south-eastern Oklahoma, USA [23]. Elshahed et al. explained that 0.7–1% salt concentration was sufficient to meet the requirements of halophilic *Archaea*. Moreover, the authors proposed the role of *Halobacteriales* in sulfur reduction and H_2_S production suggesting that halophilic *Archaea* were capable of growing under anaerobic conditions using S^0^ as a terminal electron acceptor [23]. Therefore, this microbial group might be expected in the sulfur-rich aquatic habitats, including underground waters of the Carpathian Foredeep. Although *Archaea* constituted only a small portion of microbial communities, their contribution to the underground processes in the studied waters might be still significant. Bacterial and archaeal members of the “rare biosphere” should not be neglected, and they might be even considered as “keystone species” having metabolic potential essential for subsurface ecosystems [47, 64, 65].

The chemistry of the Carpathian Foredeep sulfidic waters remaining under long-lasting monitoring (several years or longer) shows that concentrations of SO_4_^2−^ and HS^−^/H_2_S are maintained in steady state with only minor fluctuations. Abiotic reduction of sulfates to S^2−^ compounds under temperature and pressure found in the subsurface aquifers is a very slow process; thus, sulfides are expected to be generated from SO_4_^2−^ by microorganisms [66]. According to our results, the presence of HS^−^/H_2_S in the studied waters at least partially may be related to the composition and activity of microbial communities. This investigation indicated that sulfidic water samples were commonly inhabited by both the bacterial (and potentially also archaeal) sulfate reducers and sulfur oxidizers, which allow us to speculate that a complete sulfur cycle may exist in the studied subsurface aquifers. Nevertheless, the lack of correlation between HS^−^/H_2_S content and SRB/SOB number ratio suggests that more complex factor(s) than only particular group abundance may be involved in the high rate of sulfide production, e.g., cells’ metabolic activity under specific conditions or the supply of appropriate compounds used as electron donors (i.e., H_2_ and organic matter) [56, 67]. It is also likely that some part of sulfides is generated in sulfidic waters via an alternative pathway, independent of SRB. Schwermer et al. reported that desulfuration occurring during the decomposition of S-rich organic matter resulted in large H_2_S release in marine sludge, while microbial reduction of SO_4_^2−^ accounted for only 4–9% of total H_2_S [68]. Besides, the authors indicated that nitrates in concentration exceeding 0.8 mM inhibited sulfate reduction (but not sulfuration) and induced sulfide oxidation (partially to S^0^). Regarding these findings, the concentration of nitrates measured in the water of Welnin may be high enough (62.2 mg/dm^3^, ca. 1 mM) to decrease SO_4_^2−^ reduction by SRB and induce NO_3_^−^ reduction (potentially to NH_4_^+^ via dissimilatory nitrate reduction pathway) coupled with sulfide oxidation by SOB [69, 70]. It is also supported by the latest work of Lau et al., who developed a geochemical model indicating that under the subsurface conditions, sulfate reduction may be energetically a less favorable reaction compared to the redox couple of sulfur oxidation and nitrate reduction [71]. However, it is hardly probable that sulfides in Welnin originate mostly from the organic matter decomposition; therefore, SRB activity should not be neglected. Other known processes resulting in sulfide production (i.e., anaerobic methane oxidation, AOM) are unlikely explanations of huge HS^−^/H_2_S pool in Welnin, since methanotrophs were not found in this water, and what’s more, they were extremely rare in the remaining ones (from 0.02 to 0.22% of the total communities) (data not shown). Processes underlying sulfide production in other waters may be different compared to Welnin and dependent of water-specific parameters. Presumably, under much lower concentrations of both nitrates (0.55 to 0.63 mg/dm^3^, ca.8.8 to 10.1 μM; Table 1) and organic matter, the predomination of SRB-mediated process may be favored, but still the complex interplay between different pathways of sulfur transformation is expected to exist and keep the balance between sulfur forms at different oxidation states.

## Conclusions

The results presented in this paper contribute to the body of knowledge on subsurface biosphere composition. We revealed diverse bacterial and archaeal communities found in the sulfide-rich groundwaters. Dominant bacterial communities were more diverse and dissimilar than archaeal ones; however, one unclassified betaproteobacterial OTU affiliated with *Comamonadaceae* may be considered to have originated from the ancient microbiome, that could be formerly common for all studied waters. In spite of harsh environmental conditions (i.e., high concentration of HS^−^/H_2_S and low content of organic matter), the total number of cells was typical of subsurface aquifers. Raised H_2_S influenced the microbial species richness but neither the Shannon diversity nor the cell number. Among microorganisms inhabiting sulfide-rich groundwaters, the genera involved in the sulfur cycle were highly abundant; however, SOB to SRB ratio did not correlate with HS^−^/H_2_S content. Therefore, the predomination of either sulfur reducers or sulfur oxidizers might depend on water’s specific and undefined parameters.

## Electronic Supplementary Material

ESM 1 (DOCX 25 kb)

Fig. S1 Provinces of mineral and thermal waters in Poland (left panel) and location of the sampling sites (right panel). Red dots mark boreholes extracting sulfidic water. A – Precambrian Platform; B – Paleozoic Platform; C – Sudetes; D – Carpathians (consisting of the Inner Carpathians, the Outer Carpathians and the Carpathian Foredeep). (PDF 491 kb)

Fig. S2 Microscopic analysis of sulphidic waters. A – Busko 17; B – Busko 4B; C – Busko C1; D – Dobrowoda; E – Welnin. (JPG 931 kb)

Fig. S3 Principal component analysis: biplot of environmental variables and sampling sites Statistically significant variables are given in red. Variables that positively correlated with shown vectors (>0.95) are given in parentheses. (PDF 335 kb)

Fig. S4 Venn diagrams of OTUs shared at the 0.03 dissimilarity level: (A) Bacteria, (B) Archaea. (PDF 493 kb)

Fig. S5 Rarefaction curves plotting an averaged numbers of OTUs at the 0.03 dissimilarity threshold: (A) Bacteria, (B) Archaea. (PDF 389 kb)

Fig. S6 Heatmaps based on NMDS analysis and Bray-Curtis distance matrix for: (A) 100 most abundant bacterial OTUs and (B) 50 most abundant archaeal OTUs constructed at 0.03 dissimilarity level. (PDF 853 kb)

Fig. S7 Community distance heatmaps for Bacteria (upper panels) and Archaea (lower panels), based on Bray-Curtis (A,C) or Morisita-Horn dissimilarity (B,D). All heatmaps were calculated at 0.03 dissimilarity level. Lighter shades mean greater similarity. (PDF 475 kb)

Fig. S8 Non-metric multidimensional scaling plots of (A) bacterial and (B) archaeal OTUs constructed at 0.03 dissimilarity level. Square color represents sampling site, square size reflects depth of extraction on the bacterial panel or salinity on the archaeal panel. Circle color represents bacterial phylum or archaeal class, circle size correlates with OTU abundance. Statistically significant environmental variables (p<0.05) are given as red arrows. (PDF 550 kb)

Fig. S9 Non-metric multidimensional scaling plot of bacterial OTUs contributing to sulfur cycling. Square color represents sampling site, square size reflects H2S content in sulfidic water. Circle color represents bacterial groups (SOB or SRB), circle size correlates with OTU abundance. Statistically significant environmental variables (p<0.05) are given as red arrows. (PDF 174 kb)

Fig. S10 Bacterial communities structure at the (a) phylum, (b) class, (c) family and (d) genus level. (PDF 482 kb)

Fig. S11 Archaeal communities structure at the (a) phylum, (b) family and (c) genus level. (PDF 421 kb)
