# Supplementary material for: Comamonadaceae OTU as a Remnant of an Ancient Microbial Community in Sulfidic Waters
Source: Microb Ecol. 2018 Oct 19;78(1):85–101. doi: 10.1007/s00248-018-1270-5 (PMC6560000; doi:10.1007/s00248-018-1270-5)
Supplement: Supplementary file 1 — (DOCX 25 kb) [file 248_2018_1270_MOESM1_ESM.docx]

**Supplementary Data**

**Negative control: the list of “contaminating genera” removed from the dataset**

| Genus | Abundance in negative control (%) |
| --- | --- |
| *Streptococcus*  *Neisseria*  *Gemella*  *Rothia*  *Haemophilus*  *Leptotrichia*  *Granulicatella*  *Escherichia-Shigella*  *Actinomyces*  *Veilonella*  *Pseudomonas*  *Propionibacterium* | 59.4  22.3  6.5  4.2  2.3  2.1  1.2  1.0  0.4  0.2  0.1  0.1 |

The “contaminating sequences” were completely removed from the dataset because they were absent from the first series of subsamples (PCR negative controls were clean) and they appeared in the second and the third series that were prepared with the different batch of disposable plastics. Since contamination of the laboratory reagents and plastics with extraneous DNA is ubiquitous, it is crucial to have a negative control for every analysis. Therefore, amplicons from our negative controls were barcoded and sequenced along with the samples.

**Tables**

Table S1 Distances between boreholes extracting sulfidic water.

| Distance (m) | | | | |
| --- | --- | --- | --- | --- |
|  | Busko 4B | Busko 17 | Busko C1 | Dobrowoda |
| Busko 17 | 350 |  |  |  |
| Busko C1 | 2133 | 1991 |  |  |
| Dobrowoda | 7970 | 7706 | 8513 |  |
| Wełnin | 18993 | 18667 | 19194 | 11235 |

Table S2 Rare bacterial OTUs (abundance below 0.5%) shared across all water samples.

| OTU | Contribution to the bacterial diversity (%) | | | | | Taxonomical affiliation |
| --- | --- | --- | --- | --- | --- | --- |
|  | 4B | 17 | C1 | D | W |  |
| 016 | 0.30 | 2.13 | 0.06 | 2.22 | 4.07 | Bacteria; Proteobacteria; Gammaproteobacteria; unclassified |
| 023 | 0.59 | 0.55 | 0.54 | 0.39 | 0.07 | Bacteria; Proteobacteria; Alphaproteobacteria; Rhizobiales; Bradyrhizobiaceae; unclassified |
| 029 | 0.05 | 0.25 | 0.06 | 1.61 | 0.95 | Bacteria; Firmicutes; Bacilli; Bacillales; Staphylococcaceae; *Staphylococcus* |
| 037 | 0.61 | 0.23 | 0.01 | 0.08 | 0.04 | Bacteria; Candidate_division_TM7; unclassified |
| 055 | 0.06 | 0.55 | 0.01 | 0.06 | 0.68 | Bacteria; Proteobacteria; Gammaproteobacteria; Chromatiales; Halothiobacillaceae; *Halothiobacillus* |
| 068 | 0.08 | 0.39 | 0.03 | 0.12 | 0.34 | Bacteria; Firmicutes; Bacilli; Bacillales; Bacillaceae; *Bacillus* |
| 108 | 0.07 | 0.20 | 0.02 | 0.09 | 0.04 | Bacteria; Proteobacteria; Betaproteobacteria; Burkholderiales; Comamonadaceae; *Pelomonas* |

Table S3 Structure of dominant bacterial and archaeal taxa in water samples.

| Level | Taxon | Percentage (%) | | | | |
| --- | --- | --- | --- | --- | --- | --- |
|  |  | 4B | 17 | C1 | D | W |
| K | *Bacteria* |  |  |  |  |  |
| P | *Proteobacteria* | 94.43 | 88.35 | 24.38 | 77.42 | 88.79 |
| C | *Epsilonproteobacteria* | 76.92 | 3.41 | 5.92 | 2.88 | 6.41 |
| F | *Helicobacteraceae* | 71.06 | 3.38 | 5.72 | 2.76 | 5.6 |
| G | *Sulfurimonas* | **64.86** | 0.44 | 5.72 | 0.37 | 5.45 |
| G | *Sulfurovum* | 6.20 | 2.93 | 0 | 2.39 | 0.15 |
| F | *Campylobacteraceae* | 5.50 | 0 | 0.17 | 0.10 | 0.78 |
| G | *Arcobacter* | 5.49 | 0 | 0 | 0 | 0.78 |
| C | *Betaproteobacteria* | 5.55 | 49.54 | 2.90 | 45.48 | 60.96 |
| F | *Comamonadaceae* | 5.11 | 48.21 | 1.72 | 45.06 | 60.05 |
| G | *unclassified Comamonadaceae* | 5.02 | **47.25** | 1.68 | **44.41** | **57.78** |
| C | *Deltaproteobacteria* | 7.16 | 27.69 | 7.38 | 19.49 | 14.23 |
| F | *Desulfobulbaceae* | 2.63 | 24.04 | 2.64 | 8.22 | 0.21 |
| G | *Desulfopila* | 0 | **19.43** | 0 | 0 | 0 |
| G | *MSBL7* | 0.06 | 2.66 | 0.01 | 5.95 | 0 |
| G | *Desulfurivibrio* | 0.03 | 0 | 2.62 | 0.38 | 0 |
| F | *Desulfomicrobiaceae* | 0 | 0.19 | 1.39 | 0.27 | 7.44 |
| G | *Desulfomicrobium* | 0 | 0.19 | 1.39 | 0.27 | 7.44 |
| F | *Desulfovibrionaceae* | 0.02 | 0.80 | 2.30 | 5.66 | 0.04 |
| G | *unclassified Desulfovibrionaceae* | 0.02 | 0.23 | 2.26 | 1.43 | 0 |
| G | *Desulfovibrio* | 0 | 0.04 | 0.03 | 3.59 | 0.04 |
| P | *Firmicutes* | 0.40 | 1.59 | 73.55 | 3.90 | 2.44 |
| C | *Clostridia* | 0.12 | 0.67 | 72.57 | 3.32 | 1.78 |
| F | *Peptococcaceae* | 0.02 | 0.28 | 71.43 | 0.06 | 0.01 |
| G | *Candidatus Desulforudis* | 0.02 | 0.28 | **71.43** | 0.06 | 0.01 |
| K | *Archaea* |  |  |  |  |  |
| P | *Euryarcheota* | 99.5 | 99.3 | 99.0 | 99.1 | 99.9 |
| C | *Halobacteria* | 96.2 | 96.7 | 93.0 | 95.2 | 93.1 |
| F | *Halobacteriaceae* | 95.3 | 95.8 | 92.2 | 94.9 | 92.1 |
| G | *Natronomonas* | **23.3** | **31.3** | **23.6** | **28.8** | **21.2** |
| G | *Halorhabdus* | 15.6 | 15.4 | 13.4 | 14.3 | 10.5 |
| G | *unclassified Halobacteriaceae* | 13.8 | 9.4 | 14.4 | 15.4 | 16.2 |
| G | *Halorubrum* | 12.4 | 10.2 | 14.9 | 8.8 | 14.5 |
| G | *Halobellus* | 4.2 | 7.6 | 5.8 | 7.2 | 6.1 |
| G | *Haloplanus* | 6.1 | 4.5 | 4.5 | 3.0 | 4.0 |

Bold numbers indicate the dominant genera
