# Supplementary figures and images for: Comamonadaceae OTU as a Remnant of an Ancient Microbial Community in Sulfidic Waters
Source: Microb Ecol. 2018 Oct 19;78(1):85–101. doi: 10.1007/s00248-018-1270-5 (PMC6560000; doi:10.1007/s00248-018-1270-5)

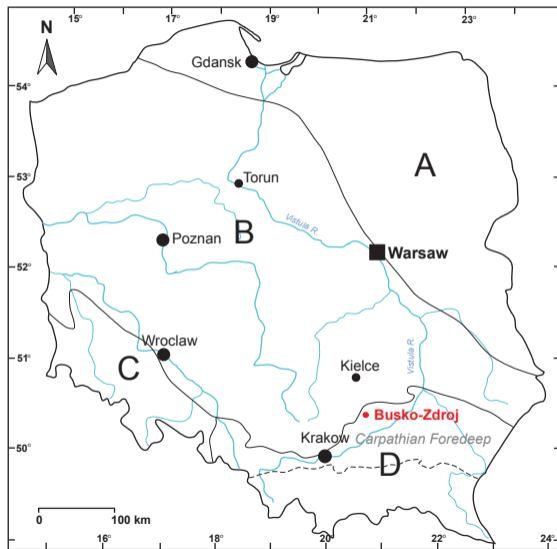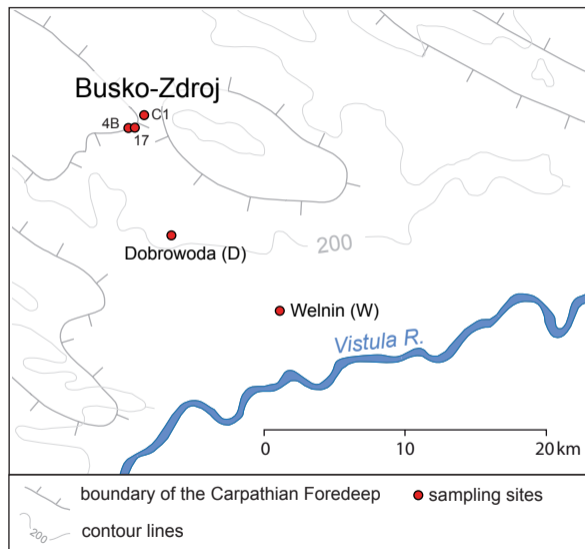

Supplement: Supplementary file 2 — Provinces of mineral and thermal waters in Poland (left panel) and location of the sampling sites (right panel). Red dots mark boreholes extracting sulfidic water. A – Precambrian Platform; B – Paleozoic Platform; C – Sudetes; D – Carpathians (consisting of the Inner Carpathians, the Outer Carpathians and the Carpathian Foredeep). (PDF 491 kb) [file 248_2018_1270_MOESM2_ESM.pdf]

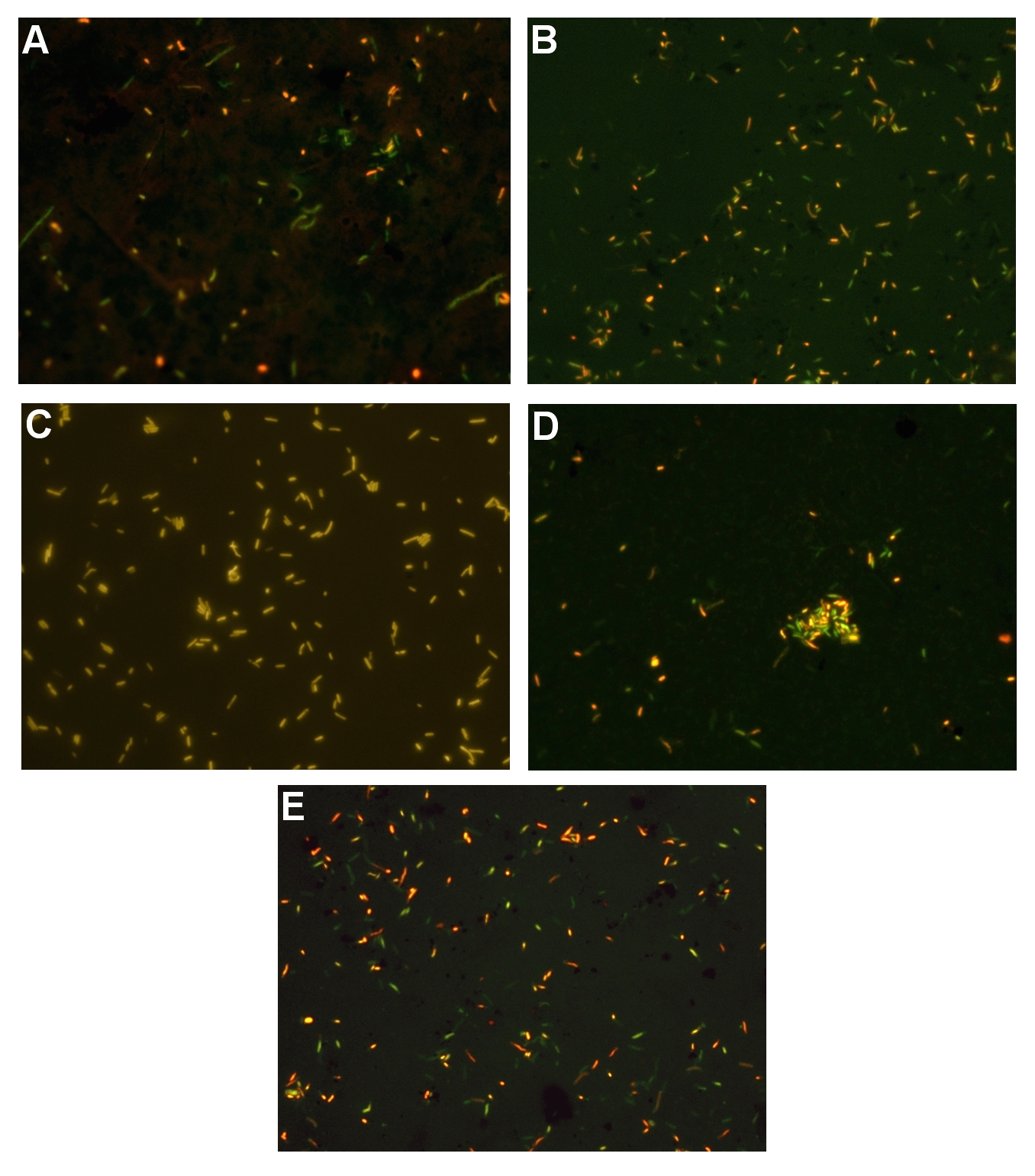

Supplement: Supplementary file 3 — Microscopic analysis of sulphidic waters. A – Busko 17; B – Busko 4B; C – Busko C1; D – Dobrowoda; E – Welnin. (JPG 931 kb) [file 248_2018_1270_MOESM3_ESM.jpg]

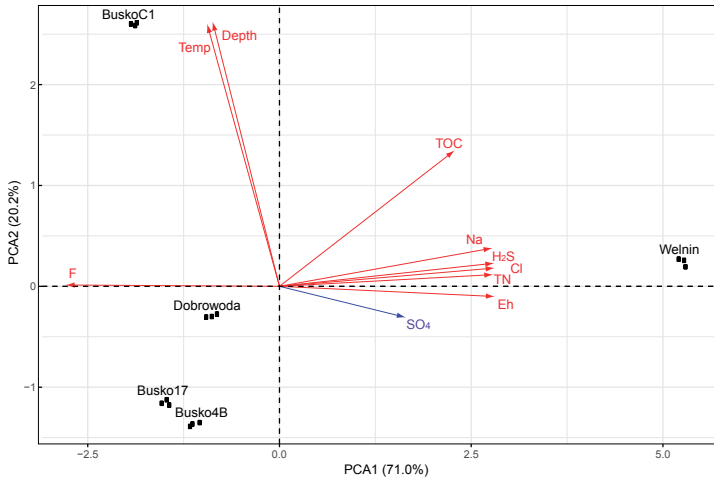

Supplement: Supplementary file 4 — Principal component analysis: biplot of environmental variables and sampling sites Statistically significant variables are given in red. Variables that positively correlated with shown vectors (>0.95) are given in parentheses. (PDF 335 kb) [file 248_2018_1270_MOESM4_ESM.pdf]

a)

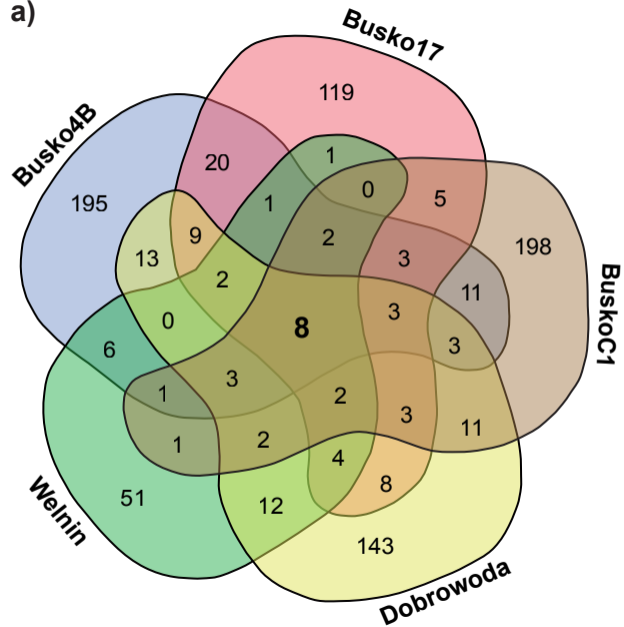

b)

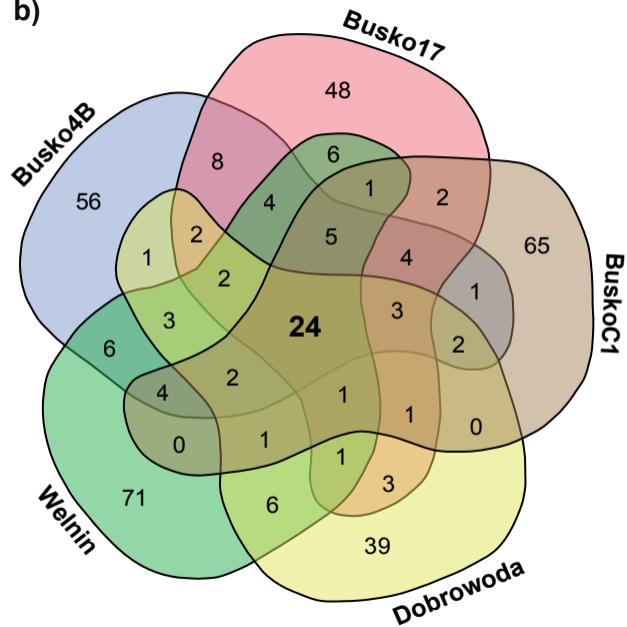

Supplement: Supplementary file 5 — Venn diagrams of OTUs shared at the 0.03 dissimilarity level: (A) Bacteria, (B) Archaea. (PDF 493 kb) [file 248_2018_1270_MOESM5_ESM.pdf]

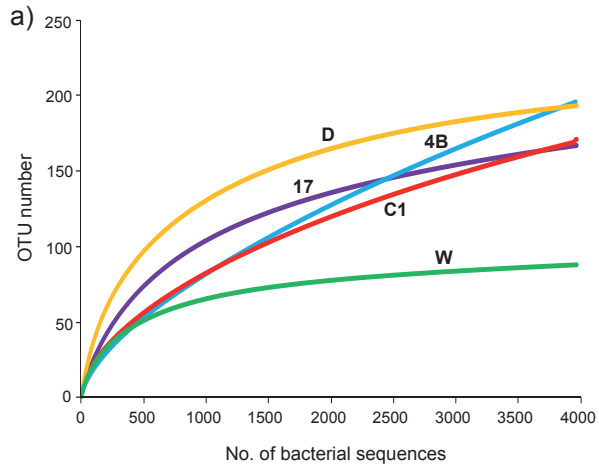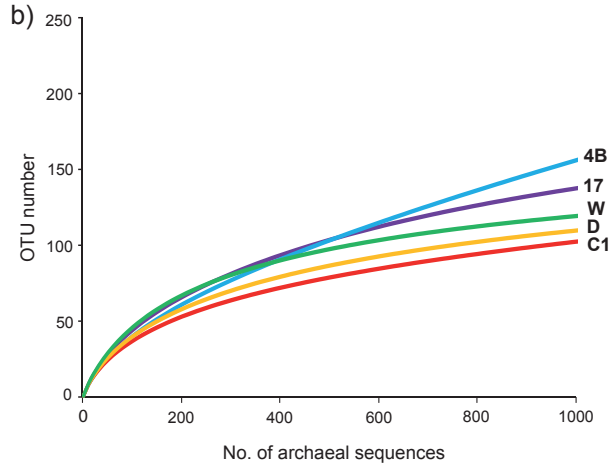

Supplement: Supplementary file 6 — Rarefaction curves plotting an averaged numbers of OTUs at the 0.03 dissimilarity threshold: (A) Bacteria, (B) Archaea. (PDF 389 kb) [file 248_2018_1270_MOESM6_ESM.pdf]

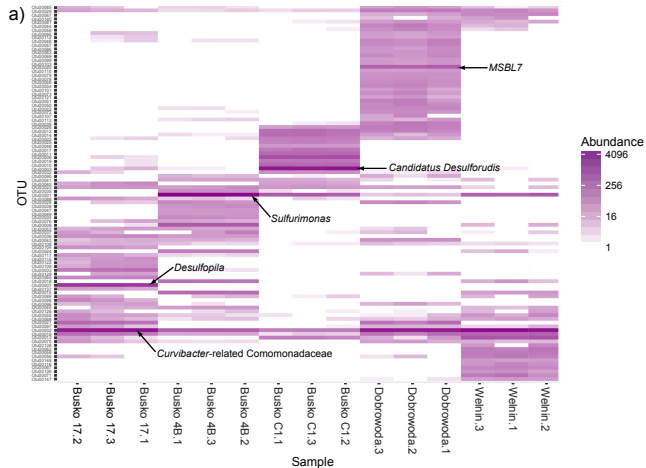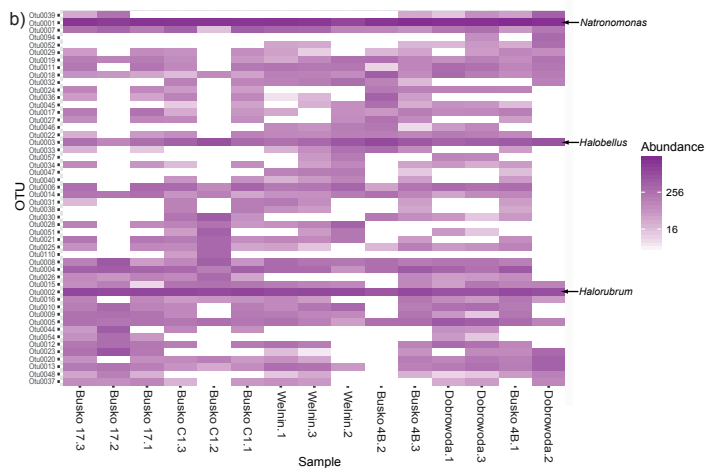

Supplement: Supplementary file 7 — Heatmaps based on NMDS analysis and Bray-Curtis distance matrix for: (A) 100 most abundant bacterial OTUs and (B) 50 most abundant archaeal OTUs constructed at 0.03 dissimilarity level. (PDF 853 kb) [file 248_2018_1270_MOESM7_ESM.pdf]

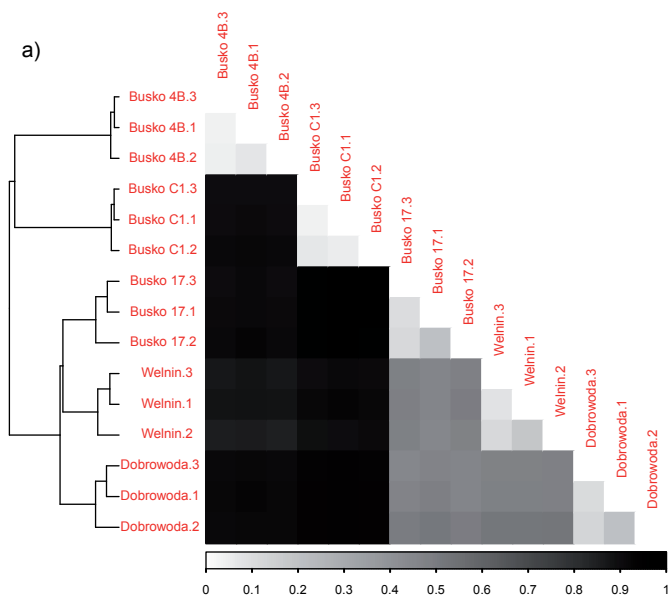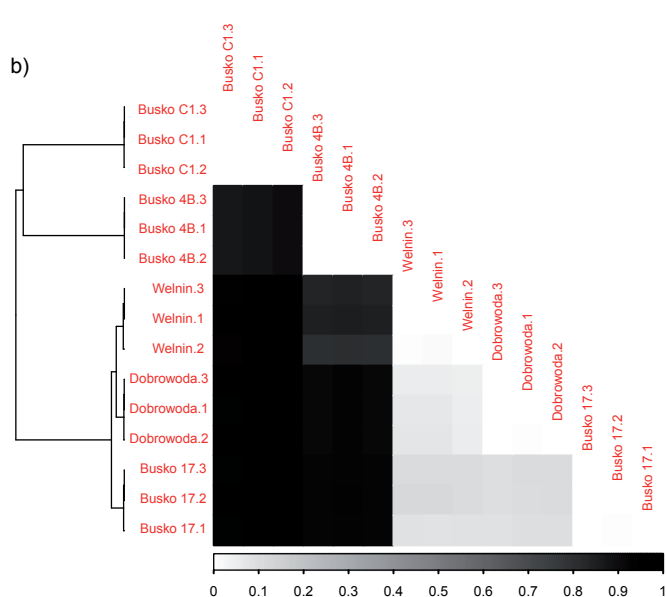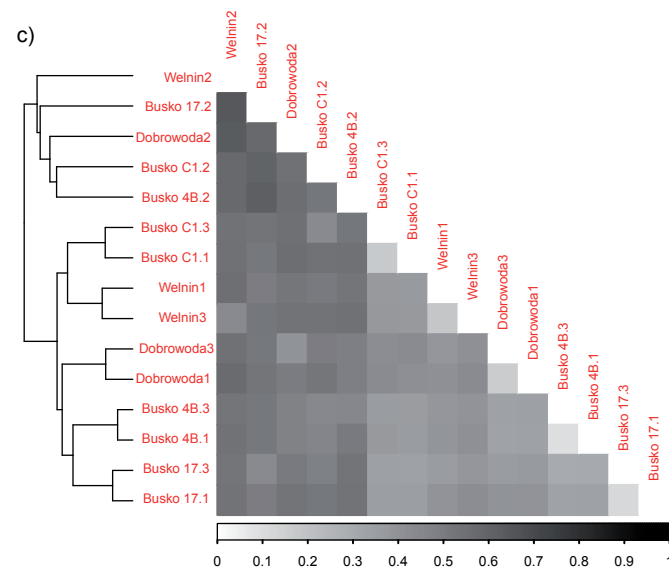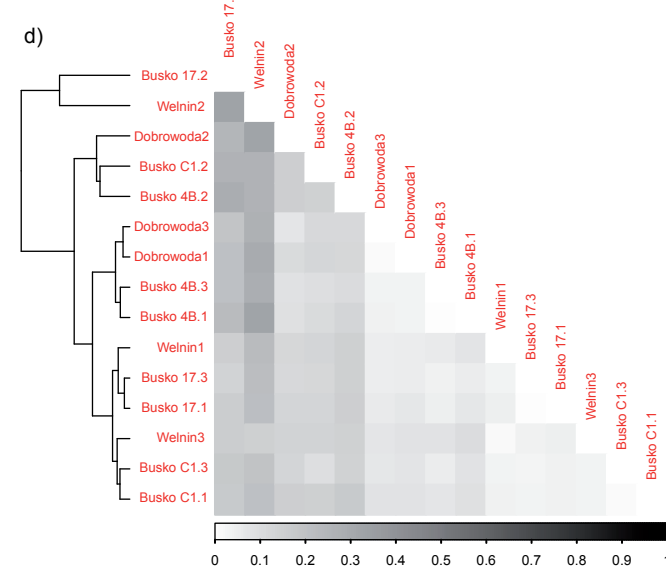

Supplement: Supplementary file 8 — Community distance heatmaps for Bacteria (upper panels) and Archaea (lower panels), based on Bray-Curtis (A,C) or Morisita-Horn dissimilarity (B,D). All heatmaps were calculated at 0.03 dissimilarity level. Lighter shades mean greater similarity. (PDF 475 kb) [file 248_2018_1270_MOESM8_ESM.pdf]

a)

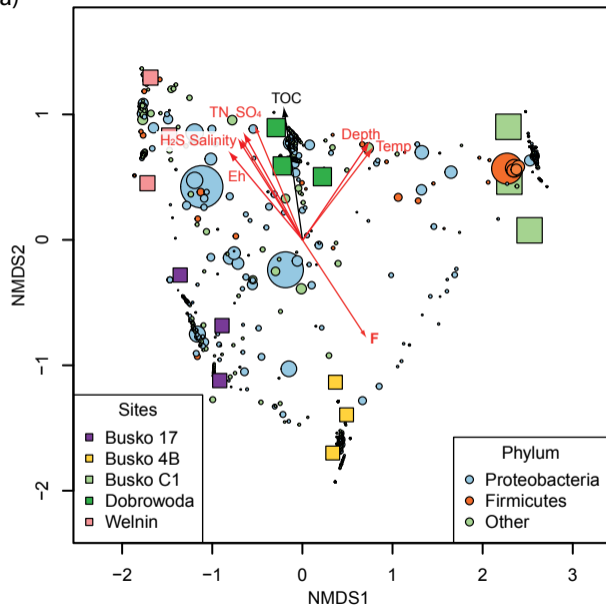

b)

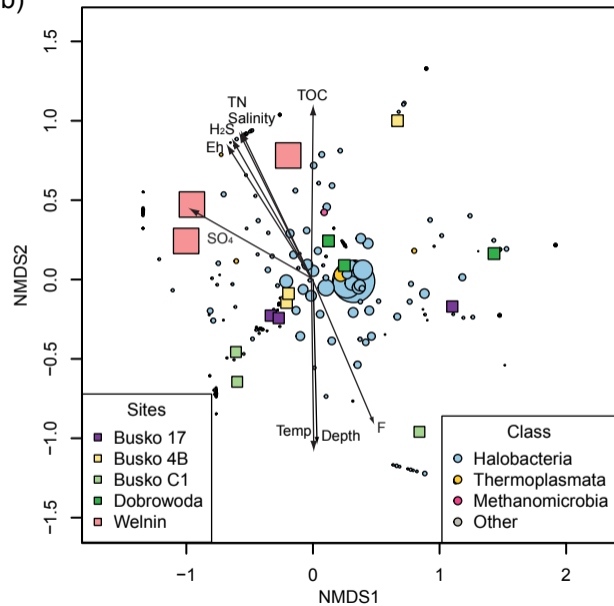

Supplement: Supplementary file 9 — Non-metric multidimensional scaling plots of (A) bacterial and (B) archaeal OTUs constructed at 0.03 dissimilarity level. Square color represents sampling site, square size reflects depth of extraction on the bacterial panel or salinity on the archaeal panel. Circle color represents bacterial phylum or archaeal class, circle size correlates with OTU abundance. Statistically significant environmental variables (p<0.05) are given as red arrows. (PDF 550 kb) [file 248_2018_1270_MOESM9_ESM.pdf]

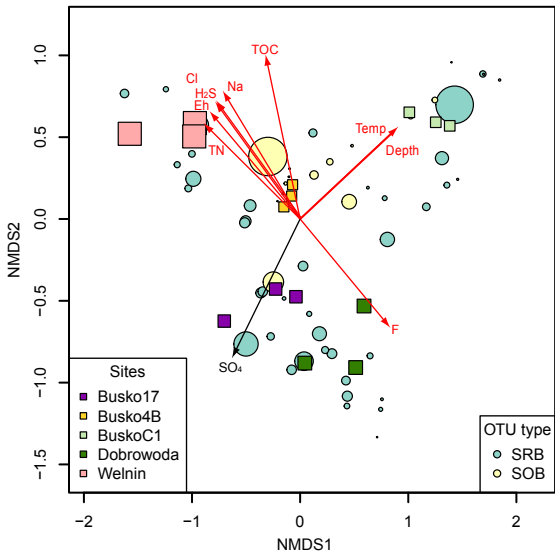

Supplement: Supplementary file 10 — Non-metric multidimensional scaling plot of bacterial OTUs contributing to sulfur cycling. Square color represents sampling site, square size reflects H2S content in sulfidic water. Circle color represents bacterial groups (SOB or SRB), circle size correlates with OTU abundance. Statistically significant environmental variables (p<0.05) are given as red arrows. (PDF 174 kb) [file 248_2018_1270_MOESM10_ESM.pdf]

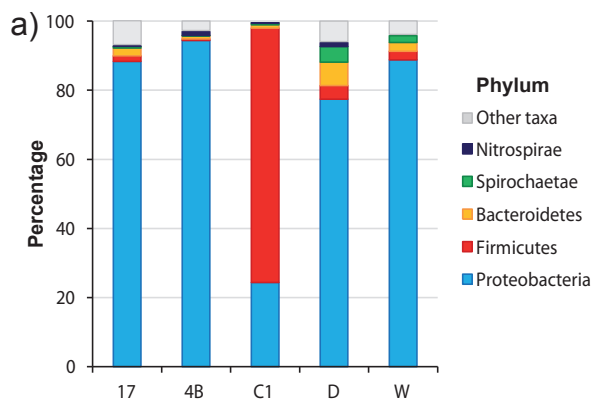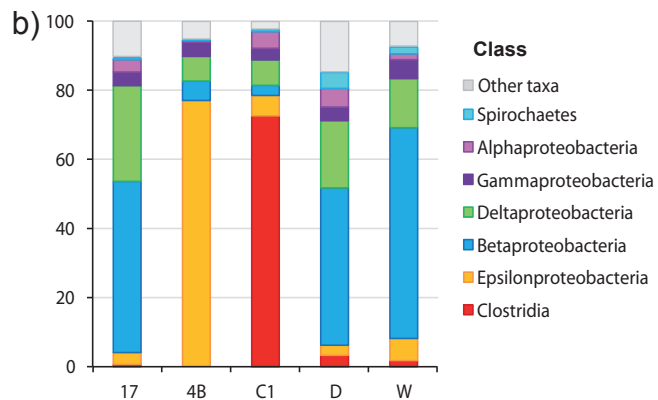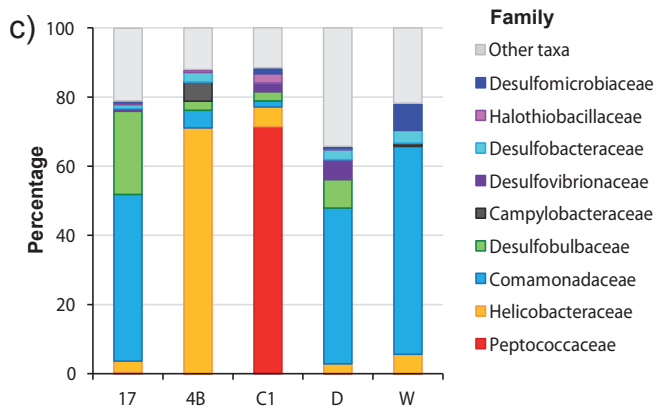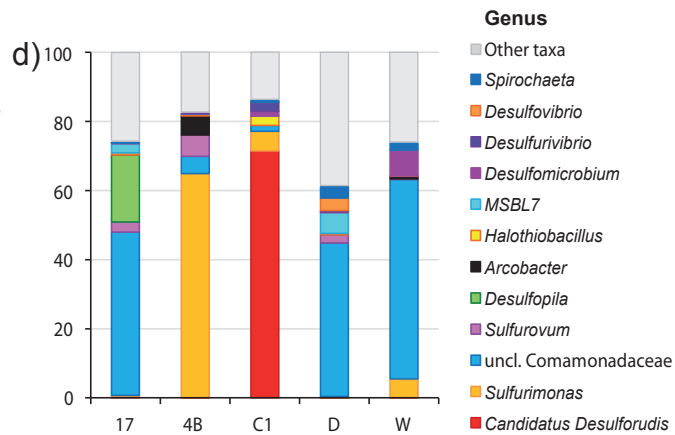

Supplement: Supplementary file 11 — Bacterial communities structure at the (a) phylum, (b) class, (c) family and (d) genus level. (PDF 482 kb) [file 248_2018_1270_MOESM11_ESM.pdf]

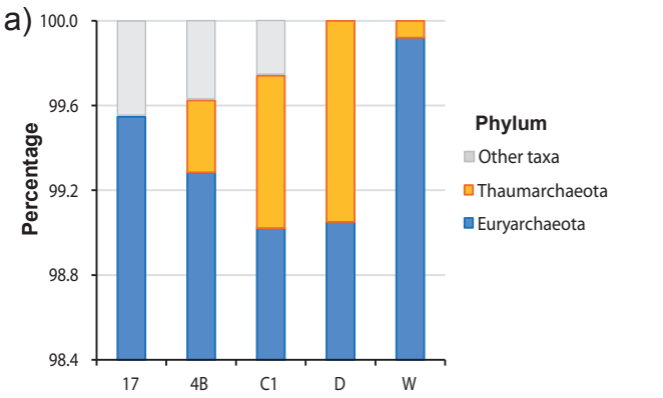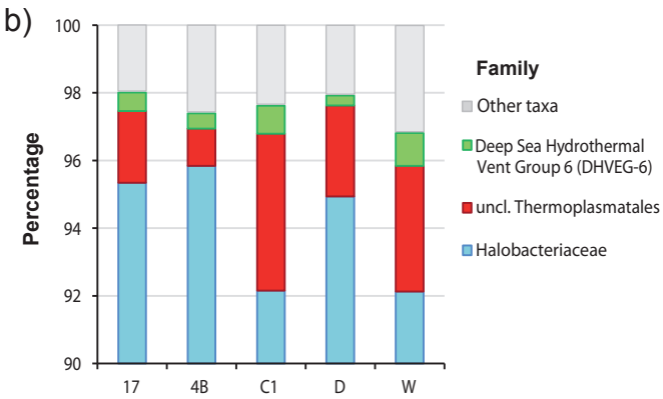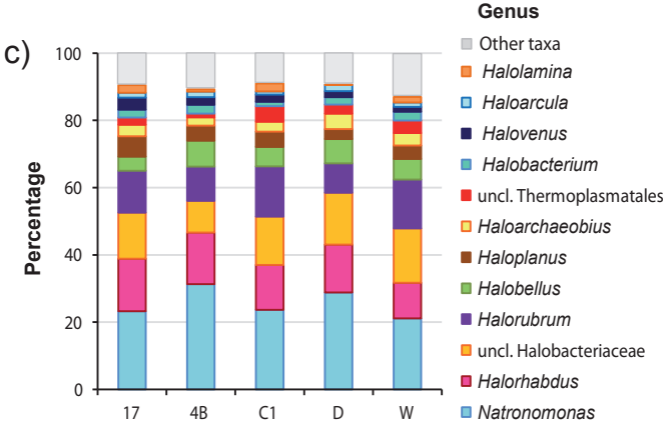

Supplement: Supplementary file 12 — Archaeal communities structure at the (a) phylum, (b) family and (c) genus level. (PDF 421 kb) [file 248_2018_1270_MOESM12_ESM.pdf]
